# Daurisoline Modulates the TBK1-Dependent Type I Interferon Pathway to Boost Anti-tumor Immunity via Targeting of LRP1

**DOI:** 10.34133/research.0764

**Published:** 2025-07-04

**Authors:** Borui Tang, Yuting Wang, Liping Li, Cuicui Sun, Jingwen Dong, Ruoqi Li, Jianfeng Wang, Yu Long, Mingxiao Yin, Fei Xie, Dian Xiao, Xinbo Zhou, Na Zhang, Xiuli Zhao, Yanchun Feng, Hongbin Deng

**Affiliations:** ^1^ School of Pharmaceutical Sciences, Capital Medical University, Beijing 100069, China.; ^2^ National Institute for Drug Clinical Trial, Beijing Tongren Hospital, Capital Medical University, Beijing 100005, China.; ^3^ Institute of Medicinal Biotechnology, Chinese Academy of Medical Sciences & Peking Union Medical College, Beijing 100050, China.; ^4^ National Engineering Research Center for the Emergency Drug, Beijing Institute of Pharmacology and Toxicology, Beijing 100850, China.; ^5^ Department of Pharmacy, Shandong Provincial Hospital Affiliated to Shandong First Medical University, Jinan 250021, China.; ^6^ National Institutes for Food and Drug Control, Beijing 102629, China.

## Abstract

A promising therapeutic approach in oncology involves immune checkpoint blockade (ICB), which stimulates anti-tumor immune responses. Nevertheless, the effectiveness of this treatment in clinical settings remains limited, underscoring the need for complementary strategies. Recent studies highlight the potential of type I interferon (IFN-I) inducers to reprogram the tumor microenvironment and enhance ICB outcomes. Herein, through high-content screening of a natural compound library, we identified daurisoline (DS), a bioactive alkaloid extracted from the Chinese herbal medicine Rhizoma Menispermi, as a potent inducer of IFN-I signaling. Our findings indicated that DS up-regulates interferon responses and pro-inflammatory cytokine expression in a TANK-binding kinase 1 (TBK1)-dependent manner. In vivo, DS exhibited marked tumor growth inhibition by activating dendritic cells, macrophages, and CD8^+^ T cells, thereby enhancing anti-tumor immunity. Utilizing the LiP-SMap approach, we identified low-density lipoprotein receptor-related protein 1 (LRP1) as the direct target of DS. Mechanistically, the binding of DS to LRP1 substantially disrupted lysosomal function, which subsequently triggered 5′-azacytidine-induced protein 2-mediated TBK1 activation and IFN-I production. Furthermore, DS demonstrated synergistic effects with anti-programmed death 1 therapy and a stimulator of interferon genes agonist by remodeling the immunosuppressive microenvironment. Collectively, our findings establish LRP1 as a novel therapeutic target for cancer immunotherapy and highlight DS-driven immune reprogramming as a translatable strategy to potentiate ICB efficacy.

## Introduction

Immune checkpoint blockade (ICB) therapies have transformed the landscape of cancer immunotherapy, emerging as powerful interventions for various cancer types [1]. Specifically, targeting key immune checkpoints—cytotoxic T lymphocyte-associated antigen 4 and programmed death 1 (PD-1)/programmed death ligand 1 (PD-L1)—releases inhibitory signals that constrain T cells’ activation, thereby eliciting robust and sustained anti-tumor immunity [2,3]. Despite these advances, the therapeutic benefits of ICB monotherapy demonstrate restricted therapeutic efficacy, benefiting only a subset of patients and specific cancer types. Hence, developing combinatorial approaches that reprogram immunologically “cold” tumors into “hot” tumors represents a critical frontier for expanding therapeutic responsiveness and enhancing the overall effectiveness of ICB therapies [4].

One promising strategy to convert immunologically “cold” tumors into “hot” ones involves the combination of ICB monotherapy with type I interferon (IFN-I) pathway activation [5]. Emerging evidence suggests that the integration of ICB therapy with IFN-I inducers can restore systemic anti-tumor immunity, thereby enhancing therapeutic efficacy across diverse malignancies and establishing durable anti-tumor responses [6–8]. IFN-I orchestrates critical immunoregulatory functions by stimulating dendritic cell (DC) maturation, augmenting CD8^+^ T-cell cytotoxicity, facilitating macrophage polarization, and directly inducing tumor cell senescence and apoptosis [8]. However, the expression of IFN-I is frequently compromised within the tumor microenvironment (TME). Consequently, identification of novel IFN-I inducers capable of enhancing tumor immunogenicity and synergizing with ICB monotherapy represents a critical strategy for improving tumor immunotherapy.

IFN-I production is triggered by pathogen-associated molecular patterns or nucleic acid fragments, including viral RNA or DNA [9]. Central to this process is TANK-binding kinase 1 (TBK1), a pivotal signaling hub for IFN-I pathway activation [10–12]. Cytoplasmic DNA is detected by specific sensors—STING (stimulator of interferon genes)—and viral RNA is recognized by MAVS (mitochondrial antiviral-signaling protein), both of which initiate TBK1 activation through induced oligomerization and autophosphorylation [13,14]. Activated TBK1 subsequently phosphorylates interferon regulator factor 3 (IRF3) and 7, driving the transcriptional activation of pro-inflammatory chemokines and the IFN-I genes [15,16]. The secreted IFN-I can then bind to its cognate receptor, the IFN-α/β receptor 1 (IFNAR1)-IFNAR2 heterodimer, initiating the JAK–STAT signaling cascade and subsequent induction of interferon-stimulated genes [17]. However, tumor-mediated silencing of these adaptor proteins often compromises IFN-I responses, underscoring the necessity for developing adaptor-independent strategies to potentiate IFN-I signaling in cancer immunotherapy.

Bioactive compounds derived from natural products or their derivatives represent a valuable resource for drug discovery and cancer treatment [18]. To date, more than 50% of small-molecule drugs approved by the Food and Drug Administration have been derived from natural products [19]. In this study, we utilized an interferon beta (IFN-β) reporter assay to screen natural compounds capable of inducing IFN-I production, leading to the identification of daurisoline (DS), a bioactive compound extracted from the Chinese herbal medicine Rhizoma Menispermi [20], as a promising and potent TBK1 activator. Our findings demonstrated that DS markedly increased pro-inflammatory cytokine expression and interferon responses through targeting low-density lipoprotein receptor-related protein 1 (LRP1). Importantly, DS demonstrated robust anti-tumor efficacy in vivo by orchestrating innate and adaptive immune responses, including activating DCs, shifting macrophage polarization, and enhancing cytotoxic CD8^+^ T-cell responses. These results highlight DS as a promising immunotherapeutic agent with potential for enhancing cancer immunotherapy outcomes.

## Results

### Discovery of DS as a potent modulator of TBK1–IFN-I signaling

**Fig. 1.**
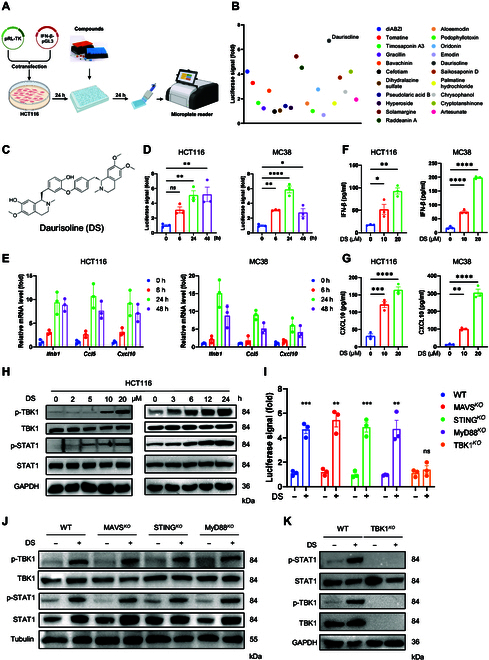
Daurisoline (DS) is a potent interferon beta (IFN-β) inducer. (A) Flow diagram of chemicals screening for potent IFN-β inducers. (B) Scatter plot of the IFN-β-Luc activity in HCT116 cells after 24-h incubation with 10 μM natural compounds. (C) Chemical structure of DS. (D and E) HCT116 or MC38 cells were treated with 20 μM DS for indicated times; the activity of IFN-β-Luc was measured by a dual-luciferase reporter gene assay (D); the messenger RNA (mRNA) expressions of *Ifnb1*, *Ccl5*, and *Cxcl10* were measured by quantitative real-time PCR (qRT-PCR) (E). (F and G) Enzyme-linked immunosorbent assay (ELISA) analysis of the IFN-β (F) and CXCL10 (G) levels in the supernatant of HCT116 and MC38 cells after treatment with 20 μM DS for 48 h. (H) HCT116 cells were treated with the indicated concentrations of DS for 24 h or treated with 20 μM DS for the indicated time points; the phosphorylation levels of TANK-binding kinase 1 (TBK1) and STAT1 were determined by immunoblotting. (I) MC38 wild type (WT), MAVS*^KO^*, STING*^KO^*, MyD88*^KO^*, or TBK1*^KO^* cells were treated with 20 μM DS for 24 h; the activity of IFN-β-Luc was measured by a dual-luciferase reporter gene assay. (J and K) MC38 WT, MAVS*^KO^*, STING*^KO^*, MyD88*^KO^*, or TBK1*^KO^* cells were treated with the indicated concentrations of DS for 24 h or treated with 20 μM DS for the indicated time points; the phosphorylation levels of TBK1 and STAT1 were determined by immunoblotting. Data are shown as the mean ± standard error of mean (SEM) of 3 independent experiments. Statistical significance was analyzed by an unpaired Student *t* test (D to G and I). *P < 0.05; **P < 0.01; ***P < 0.001; ns, not significant. GAPDH, glyceraldehyde-3-phosphate dehydrogenase.

Given the pivotal role of IFN-I signaling in tumor immunity, a natural bioactive chemical library was screened (Selleck L1400) using an IFN-β-Luc reporter assay to identify potential IFN-I inducers (Fig. 1A). The screening results were visualized in a scatter plot, illustrating the relative change in luciferase signal responses to the various chemicals (Fig. 1B). Notably, DS, a natural compound extracted from the Chinese herbal medicine Rhizoma Menispermi [20], demonstrated superior IFN-β promoter activation in our screening assay (Fig. 1B and C). We then assessed the cytotoxicity of DS on cancer cells. DS exhibited selective cytotoxicity, with IC_50_ values of 29.4 and 34.6 μM for HCT116 and MC38 cells, respectively, which were markedly lower than its toxicity toward normal cells (Fig. S1A and B).

To further clarify the involvement of DS in the activation of IFN-I signaling, we treated tumor cells with DS and observed a robust enhancement in IFN-β-Luc activity (Fig. 1D) in both HCT116 and MC38 cells, exhibiting a time- and dose-dependent response. Additionally, real-time polymerase chain reaction (PCR) analysis revealed that DS treatment substantially up-regulated the expression of genes associated with IFN-I signaling, including *Ifnb1*, *Ccl5*, and *Cxcl10*, in HCT116 and MC38 cells (Fig. 1E). Furthermore, enzyme-linked immunosorbent assay (ELISA) results indicated that DS treatment markedly elevated the secretion of downstream cytokines in the IFN-I pathway, including IFN-β and CXCL10 (Fig. 1F and G). At the molecular level, DS dose- and time-dependently enhanced the phosphorylation of key IFN-I signaling mediators, TBK1 and STAT1, in HCT116 cells (Fig. 1H), with consistent activation patterns observed in SW620 and MC38 cells (Fig. S1C and D). These results collectively reinforce the conclusion that DS activates the IFN-I pathway.

It is well established that TBK1 adapter proteins, including MAVS, STING, and MyD88, play crucial roles in regulating IFN-I production [21,22]. To investigate whether these adaptor proteins mediate DS-induced IFN-I activation, we genetically deleted MAVS, STING, and MyD88. Notably, DS-induced IFN-I activation remained intact in these knockout models, as evidenced by sustained IFN-β-Luc activity and TBK1/STAT1 phosphorylation (Fig. 1I and J). In contrast, TBK1 deficiency substantially attenuated DS-mediated IFN-I signaling (Fig. 1I and K). Together, these findings identify DS as an effective IFN-I pathway inducer that modulates this pathway through a TBK1-dependent mechanism.

### DS-treated tumor cells promote DC maturation, macrophage polarization, and T-cell activation

**Fig. 2.**
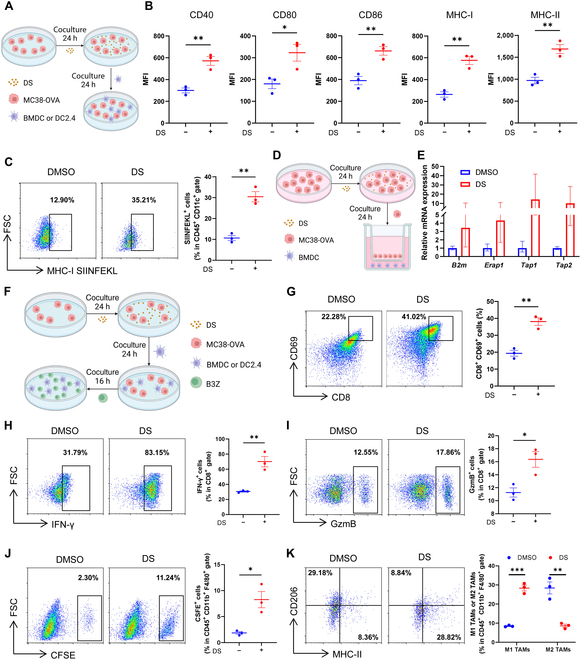
DS-treated tumor cells promote dendritic cell (DC) maturation, macrophage polarization, and T-cell activation. (A to C) MC38-ovalbumin (OVA) cells were treated with dimethyl sulfoxide (DMSO) or DS (20 μM) for 24 h and then cocultured with bone-marrow-derived DCs (BMDCs) for an additional 24 h (A), after which the surface expressions of CD40, CD80, CD86, MHC-I, MHC-II (B), and MHC-I-SIINFEKL (C) on BMDCs were determined by flow cytometry analysis. (D and E) MC38-OVA cells were treated with DMSO or DS (20 μM) for 24 h, followed by transferring to a transwell coated with BMDCs and coculturing for an additional 24 h (D); the mRNA levels of antigen processed genes *B2m*, *Erap1*, *Tap1*, and *Tap2* in BMDCs were detected by qRT-PCR (E). (F to I) MC38-OVA cells were treated with DMSO or DS (20 μM) for 24 h, followed by coculturing with BMDCs for 24 h and B3Z cells for an additional 16 h (F), and then the surface expression of CD69 (G) and the secretion of effector molecules interferon gamma (IFN-γ) (H) and granzyme B (GzmB) (I) sin B3Z cells was examined by flow cytometry analysis. (J) Carboxyfluorescein succinimidyl ester (CFSE)-stained MC38 cells were treated with DMSO or DS (20 μM) for 24 h, followed by coculturing with bone-marrow-derived macrophages (BMDMs) for an additional 24 h; the proportion of CFSE^+^ cells in CD11b^+^ F4/80^+^ macrophages was monitored by flow cytometry analysis. (K) MC38 cells were treated with DMSO or DS (20 μM) for 24 h and then cocultured with BMDMs for an additional 24 h; the proportions of MHC-II^+^ cells and CD206^+^ cells in CD11b^+^ F4/80^+^ macrophages were monitored by flow cytometry analysis. The experiments were repeated 3 times independently with similar results. Data are shown as the mean ± SEM of 3 independent experiments. *P < 0.05; **P < 0.01; ***P < 0.001. Statistical significance was analyzed by an unpaired Student *t* test (B, C, E, G, and H to K). MFI, mean fluorescence intensity; FSC, forward scatter; TAMs, tumor-associated macrophages.

As IFN-I modulates the host’s anti-tumor immune response by activating DCs and macrophages and enhancing T-cell cytotoxicity [6], we subsequently investigated the immunomodulatory effects of DS-treated tumor cells. Using an MC38-ovalbumin (OVA) and bone-marrow-derived DC (BMDC) coculturing model (Fig. 2A), we observed that DS-treated MC38-OVA cells markedly promoted the maturation of BMDCs, as indicated by the up-regulation of the surface expression levels of CD40, CD80, CD86, MHC-I, and MHC-II (Fig. 2B). Comparable results were obtained with DC2.4 (Fig. S2A). Furthermore, DS treatment markedly boosted SIINFEKL (OVA amino acids 257 to 264)-MHC-I complex expression on both BMDCs (Fig. 2C) and DC2.4 (Fig. S2B), suggesting that DS-treated tumor cells enhance DC antigen presentation capacity. Specifically, DS treatment specifically up-regulated key antigen processing and presentation genes in BMDCs: MHC-I subunit *B2m*, peptide-trimming enzyme *Erap1*, and the *Tap1*/*Tap2* transporter system required for peptide loading (Fig. 2D and E), further supporting enhanced antigen presentation. Next, we assessed the potential of DS-treated MC38-OVA cells to activate CD8^+^ T cells. Following 24-h treatment of MC38-OVA cells with DS or dimethyl sulfoxide (DMSO), cells were sequentially cocultured with BMDCs for 24 h and cocultured with OVA-epitope-specific B3Z CD8^+^ T cells for 16 h (Fig. 2F). As anticipated, B3Z cells cocultured with DS-treated MC38 cells exhibited robust up-regulation in the expression of the early activation marker CD69 (Fig. 2G). Additionally, the effector molecules including interferon gamma (IFN-γ; Fig. 2H) and granzyme B (GzmB) (Fig. 2I) were dramatically increased following coculture. Comparable results were observed when DC2.4 was utilized in place of BMDCs (Fig. S2C to E). Given that M1-polarized tumor-associated macrophages (TAMs) exhibit tumor-killing activity, whereas M2-polarized TAMs suppress anti-tumor immunity and promote tumor growth [23,24], we assessed the impact of DS on macrophage polarization. When bone-marrow-derived macrophages (BMDMs) were incubated with DS-treated MC38 cells (Fig. S2F), macrophage phagocytosis was greatly enhanced (Fig. 2J). Consistently, DS-treated MC38 cells up-regulated the levels of M1-associated costimulatory molecules, including CD86 and MHC-II, while markedly down-regulating CD206, a surface marker of M2 macrophages (Fig. 2K and Fig. S2G), indicating that DS-treated tumor cells polarize macrophages. Collectively, these findings demonstrate that DS-treated tumor cells can enhance the maturation of DCs, the activation of CD8^+^ T cells, and the polarization of macrophages toward an anti-tumor phenotype.

### DS suppresses tumor growth via activating anti-tumor immunity

**Fig. 3.**
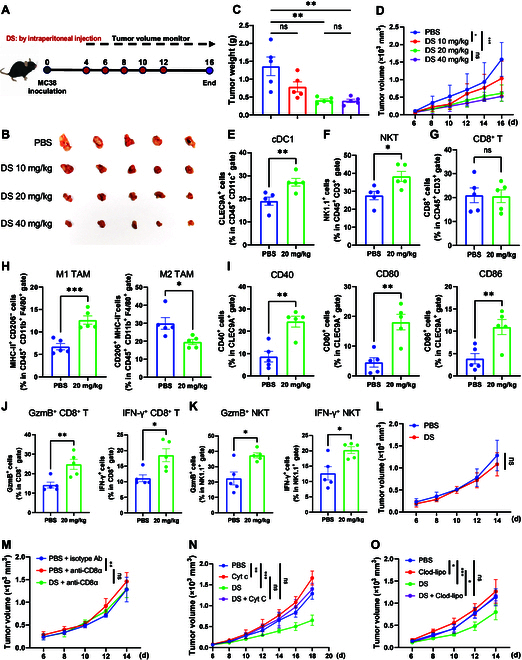
DS suppresses tumor growth and stimulates anti-tumor immunity. (A to D) C57BL/6 mice with subcutaneous MC38 tumor (*n* = 5) were intraperitoneally injected with phosphate-buffered saline (PBS) or DS (10, 20, and 40 mg/kg) (A), representative tumor images in mice are shown (B), and the tumor weight (C) and tumor volume (D) were measured for 16 d. (E to K) The proportion of type I conventional dendritic cell (cDC1s) (E), natural killer T (NKT) cells (F), CD8^+^ T cells (G), and M1 and M2 TAMs (H); the proportion of the surface expression levels of CD40, CD80, and CD86 on cDC1s (I); and the proportion of effector molecules GzmB^+^ and IFN-γ^+^ cells in CD8^+^ T cells (J) and NKT cells (K) in the tumor microenvironment (TME) from the PBS and 20 mg/kg groups were measured by flow cytometry analysis. (L) Balb/c-null mice with subcutaneous MC38 tumor (*n* = 6) were intraperitoneally injected with PBS or DS (20 mg/kg); the tumor volume was measured for 14 d. (M) C57BL/6 mice bearing MC38 tumor (*n* = 6) were treated with PBS, an anti-CD8α neutralizing antibody, or an isotype antibody (100 μg per mouse, intravenous, −1, 4, 6, 8, and 10 d) and/or DS (20 mg/kg, intraperitoneal); the tumor volume was measured for 14 d. (N) C57BL/6 mice bearing MC38 tumor (*n* = 6) were treated with PBS, cytochrome c (Cyt c; 5 mg per mouse, intravenous, −1, 7, and 14 d) that depletes DCs, and/or DS (20 mg/kg, intraperitoneal); the tumor volume was monitored for 18 d. (O) C57BL/6 mice bearing MC38 tumor (*n* = 6) were treated with PBS, clodronate liposomes (clod-lipo) (−1, 5, and 10 d) that deplete macrophages, and/or DS (20 mg/kg, intraperitoneal); the tumor volume was monitored for 14 d. Data are presented as mean ± SEM. Statistical significance was analyzed by 1-way analysis of variance (ANOVA) test (C), unpaired Student *t* test (E to K), or 2-way ANOVA test (D and L to O). *P < 0.05; **P < 0.01; ***P < 0.001; ns, not significant.

To validate the anti-tumor efficacy of DS in vivo, MC38 cells were subcutaneously inoculated into C57BL/6 mice, which were then treated with intraperitoneal injections of phosphate-buffered saline (PBS) or DS every other day for 16 d (Fig. 3A). DS treatment at dosages of 10, 20, and 40 mg/kg led to marked inhibition of tumor growth in MC38-tumor-bearing mice, achieving inhibition rates of 61.1% at 20 mg/kg and 66.9% at 40 mg/kg tumor (Fig. 3B). Furthermore, DS treatment at 20 and 40 mg/kg led to a pronounced decrease in both tumor volume and weight compared to that in the PBS group (Fig. 3C and D). Notably, DS treatment did not remarkably affect mouse body weight (Fig. S3A) or alter serum biochemical parameters, including alanine aminotransferase, aspartate aminotransferase, creatinine, and α-hydroxybutyrate dehydrogenase (Fig. S3B). Additionally, no detectable changes were observed in organ indices or histological conditions (inflammation, necrosis, or structural abnormalities) in the heart, liver, spleen, lung, and kidney (Fig. S3C and D), suggesting that DS lacks appreciable systemic toxicity in mice.

Analysis of the TME demonstrated that DS treatment substantially increased the infiltration of tumor-infiltrating type I conventional dendritic cells (cDC1s), identified as CD103^+^ CD11c^+^ DCs, and natural killer T (NKT) cells (NK1.1^+^ CD3^+^ CD45^+^) (Fig. 3E and F). While the population of tumor-infiltrating CD8^+^ T cells was only minimally affected (Fig. 3G), DS treatment markedly increased the population of M1-polarized macrophages (MHC-II^+^ CD206^−^) and decreased the proportion of M2-polarized TAMs (MHC-II^−^ CD206^+^) (Fig. 3H), suggesting a shift toward a pro-inflammatory, tumor-suppressive phenotype. In line with in vitro findings, tumor-infiltrating cDC1s exhibited elevated expression levels of CD40, CD80, and CD86 following DS treatment (Fig. 3I). Furthermore, DS treatment dramatically boosted the production of effector molecules, GzmB and IFN-γ, secreted by CD8^+^ T cells and NKT cells within the TME (Fig. 3J and K), implying that DS promotes the activation of these immune cells in vivo.

In BALB/c nude mice lacking functional T cells, DS exhibited no appreciable anti-tumor activity against MC38 tumor (Fig. 3L and Fig. S4A and B). The importance of T cells in DS-mediated anti-tumor effects was further corroborated by the finding that CD8^+^ T-cell depletion, achieved via a CD8-neutralizing antibody, abrogated DS’s anti-tumor efficacy (Fig. 3M and Fig. S4C and D). To investigate the involvement of DCs and macrophages in the anti-tumor effects of DS, we used cytochrome c (Cyt c) [25] and clodronate liposomes [26] to deplete these cell types. Intravenous administration of Cyt c or clodronate liposomes effectively depleted splenic DCs or macrophages in treated mice (Fig. S4E and F). As expected, depletion of either DCs (Fig. 3N and Fig. S4G and H) or macrophage negated the inhibitory effect of DS on MC38 tumor growth (Fig. 3O and Fig. S4I). These findings collectively suggest that DS exerts its anti-tumor activity by promoting DC maturation, enhancing T-cell cytotoxicity, and inducing macrophage polarization toward an anti-tumor phenotype.

### DS induces IFN-I response through inhibition of lysosomal activity

**Fig. 4.**
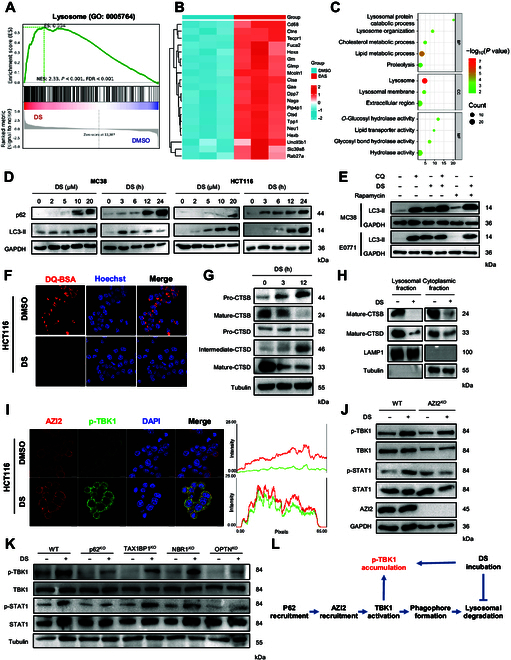
DS-mediated lysosomal inhibition induces type I interferon (IFN-I) activation. (A) According to RNA sequencing (RNA-seq) data, gene set enrichment analysis (GSEA) enriched DS into Gene Ontology (GO) lysosome. NES, normalized enrichment score; NOM, nominal; FDR, false discovery rate. (B) Lysosome-related genes (GO: 0005764) differentially expressed in MC38 cells treated with DMSO or DS (20 μM) analyzed by RNA-seq data and shown in the heatmap. (C) Plots of GO biological process enrichment showed a positive correlation with the lysosome-related pathway after treatment by DS in MC38 cells. (D) MC38 and HCT116 cells were treated with DS at the indicated concentrations for 24 h or treated with DS (20 μM) for the indicated time points; the levels of p62/SQSTM1 and LC3-II were detected by immunoblotting. (E) MC38 and E0771 cells were pretreated with chloroquine (CQ; 50 μM) or rapamycin (200 nM) for 2 h, followed by DS (20 μM) treatment for 12 h. The levels of LC3-II in both cells were detected by immunoblotting. (F) HCT116 cells were treated with DS (20 μM) for 6 h and incubated with DQ-Red BSA (10 μg/ml) for 2 h. The cells were fixed and analyzed for fluorescence microscopy (scale bars, 10 μm). (G) HCT116 cells were treated with 20 μM DS for 3 or 12 h; the precursor and the mature form of cathepsin B (CTSB) and cathepsin D (CTSD) were determined by immunoblotting. (H) Immunoblotting analysis of the cytosolic and lysosomal distributions of mature CTSB and CTSD levels in HCT116 cells treated with 20 μM DS for 12 h. Lysosomal-associated membrane protein 1 (LAMP1) was used as a lysosomal marker; tubulin was used as the cytosolic marker. (I) Immunofluorescence analysis of the colocalization between phosphorylated TBK1 (p-TBK1) and 5′-azacytidine-induced protein 2 (AZI2) in HCT116 cell treatment with DS (20 μM) for 6 h (scale bars, 10 μm). The intensity profiles of p-TBK1 and AZI2 are shown in the right panel. (J) HCT116 WT and AZI2*^KO^* cells were treated with DMSO or DS (20 μM) for 24 h; the phosphorylation levels of TBK1 and STAT1 were analyzed by immunoblotting. (K) HCT116 WT, p62*^KO^*, TAX1BP1*^KO^*, NBR1*^KO^*, and OPTN*^KO^* cells were treated with DMSO or DS (20 μM) for 24 h; the phosphorylation levels of TBK1 and STAT1 were analyzed by immunoblotting. (L) Flowchart showing the key events related to DS-mediated TBK1 activation. DS inhibits lysosome activity and leads to p-TBK1 accumulation induced by inhibition of lysosomal degradation. BP, Biological Process; CC, Cellular Component; MF, Molecular Function; DAPI, 4′,6-diamidino-2-phenylindole.

To elucidate the mechanism underlying DS-triggered IFN-I activation, RNA sequencing analysis was conducted on DS-treated MC38 cells. Interestingly, gene set enrichment analysis (GSEA) identified a pronounced enrichment of genes associated with lysosomal signaling (Gene Ontology [GO]: 0005764) in response to DS treatment (Fig. 4A). Among the enriched pathways, several lysosome-related genes, including *Mcoln1*, *Ctsa*, *Ctsd*, and *Tpp1*, were markedly up-regulated (Fig. 4B). Furthermore, this observation was supported by GO enrichment analysis of differentially expressed genes (*q* < 0.05, fold change > 2), which also highlighted the prominent role of lysosomal genes (Fig. 4C). Concurrently, GSEA demonstrated robust enrichment of the IFN-β signaling pathway (GO: 0035458) (Fig. S5A), further validating DS-mediated activation of IFN-I responses (Fig. S5B). Given that an acidic pH is essential for lysosomal activity, we utilized LysoSensor Green dye staining to assess whether DS treatment influenced lysosomal pH. The results showed no appreciable change in green fluorescence intensity, suggesting that DS does not inhibit lysosomal acidification (Fig. S5C). To further investigate lysosomal activity, we observed a concentration- and time-dependent accumulation of p62/SQSTM1 and LC3-II in both HCT116 and MC38 cells following DS treatment (Fig. 4D). Moreover, the LC3-II turnover assay [27] showed that DS-mediated LC3-II accumulation was insensitive to chloroquine but was enhanced by rapamycin cotreatment (Fig. 4E). This finding was further supported by reduced DQ-Red-BSA dequenching (Fig. 4F) and increased yellow puncta formation in GFP-LC3/RFP-LC3 assays in HCT116 cells (Fig. S5D). Immunoblotting (IB) analysis confirmed that DS substantially impaired the maturation of cathepsin B (CTSB) and cathepsin D (CTSD) in both HCT116 (Fig. 4G) and MC38 cells (Fig. S5E). Furthermore, assays of lysosomal and cytoplasmic fractions revealed that DS abrogated the maturation of CTSB and CTSD within lysosomes (Fig. 4H and Fig. S5F), indicating that DS acts as a late-stage autophagy inhibitor by impairing lysosomal function.

To investigate how DS-mediated lysosomal dysfunction triggers TBK1 activation, we considered a previous finding that the aggregation of selective autophagy cargo receptors precedes 5′-azacytidine-induced protein 2 (AZI2) recruitment and TBK1 activation [28]. Immunofluorescence analysis indicated that DS treatment facilitated the recruitment of AZI2 and phosphorylated TBK1 (p-TBK1) to form a punctate structure (Fig. 4I). Furthermore, CRIPSR-mediated depletion of AZI2 abolished DS-mediated activation of TBK1 and STAT1 (Fig. 4J), highlighting the critical role of AZI2 in DS-induced TBK1 activation. To identify the specific selective autophagy cargo receptor involved, we genetically knocked down *p62*, *TAX1BP1*, *NBR1*, and *OPTN*. Silencing of *p62* and *OPTN* remarkably attenuated DS-mediated phosphorylation of TBK1 and STAT1 (Fig. 4K). Collectively, these findings suggest that DS-mediated lysosomal dysfunction induces AZI2 recruitment in a p62- and OPTN-dependent manner, ultimately leading to TBK1 activation (Fig. 4L).

### DS inhibits lysosomal activity through directly binding to LRP1

**Fig. 5.**
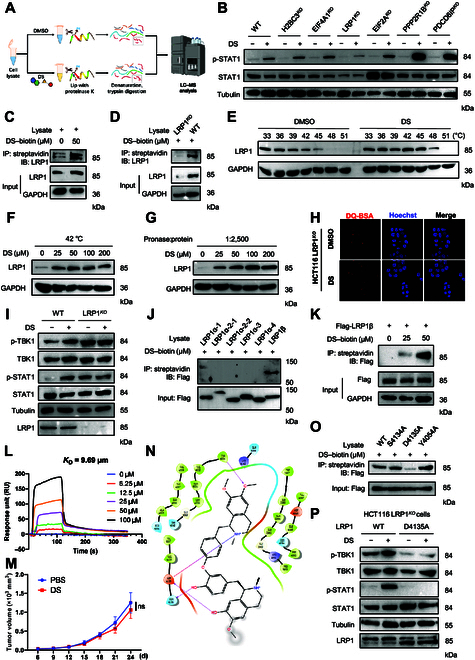
Low-density lipoprotein receptor-related protein 1 (LRP1) is the direct target protein of DS. (A) Flowchart depicting the LiP-MS assay. Freshly prepared whole-cell lysates were treated with or without DS, followed by proteinase K digestion and trypsin digestion and analyzed by liquid chromatography–mass spectrometry (LC–MS). (B) Immunoblotting analysis of the phosphorylation level of STAT1 in MC38 cells with the indicated genes’ knockout after treatment with DMSO or DS (20 μM) for 24 h. (C and D) Immunoblotting analysis of streptavidin–agarose-precipitated LRP1 from HCT116 cells (C), MC38 LRP1*^KO^* cells, or MC38 WT cells (D) incubated with DS–biotin at 4 °C overnight. (E) Cellular thermal shift assay (CETSA) analysis of the binding of DS to LRP1 in HCT116 cells, with GAPDH serving as the internal control. (F) The stability of LRP1 engagement by the indicated concentrations of DS at 42 °C was determined by CETSA. (G) Drug affinity responsive target stability (DARTS) assay determining the stability of LRP1 engagement by the indicated concentrations of DS when the ratio of Pronase to protein was 1:2,500 in HCT116 cells. (H) HCT116 LRP1*^KO^* cells were treated with DS (20 μM) for 6 h before being incubated with DQ-Red BSA (10 μg/ml) for 2 h. Red fluorescence stimulated by green laser was detected by flow cytometry analysis. The cells were fixed and analyzed for fluorescence microscopy (scale bars, 10 μm). (I) Immunoblotting analysis of the phosphorylation levels of TBK1 and STAT1 in MC38 WT cells and LRP1*^KO^* cells treated with DS (20 μM) for 24 h. (J) Coimmunoprecipitation assay mapping of LRP1 domains critical for DS binding. (K) Coimmunoprecipitation assay determination of the binding of the indicated concentrations of DS to recombinant LRP1β. (L) Surface plasmon resonance (SPR) analysis of DS and LRP1β binding. An activated anti-His tag sensor chip was used to immobilize the recombinant human LRP1β protein and flowed across DS. (M) C57BL/6 mice with subcutaneous LRP1*^KO^* MC38 tumor (*n* = 6) were intraperitoneally injected with PBS or DS (20 mg/kg). The tumor volume was measured for 24 d. (N) Molecular docking model revealing how DS binds to the β domain of LRP1. (O) Coimmunoprecipitation assay determination of the binding of DS to LRP1 WT, S4134A, D4135A, or Y4054A. (P) Immunoblotting analysis of the phosphorylation levels of TBK1 and STAT1 in HCT116 LRP1*^KO^* cells transfected with LRP1 WT or LRP1-D4135A plasmids for 48 h. Data are presented as mean ± SEM. Statistical significance was analyzed by 2-way ANOVA test (M). *P < 0.05; **P < 0.01; ***P < 0.001; ns, not significant. IP, immunoprecipitation; IB, immunoblotting.

To identify the potential targets of DS associated with the IFN-I pathway, we utilized the LiP-SMap [29] technique (Fig. 5A). The top candidates, characterized by a Delta-preference peptide >5 and a preference full-trypsin peptide ratio <0.5, are listed in Fig. S6A. Interestingly, CRISPR-mediated knockout of LRP1, a member of the low-density lipoprotein receptor family, specifically abrogated DS-induced STAT1 activation (Fig. 5B), indicating that LRP1 is a DS-interacting protein. To confirm this interaction, we developed a DS probe by conjugating DS with biotin via a propyl linker. Following verification that DS–biotin retained its capability of inducing STAT1 activation and IFN-β-Luc activity (Fig. S6B and C), we incubated DS–biotin with MC38 or HCT116 cell lysates. A biotin–streptavidin affinity pull-down assay confirmed the interaction between DS and LRP1 in both MC38 and HCT116 cell lysates (Fig. 5C and Fig. S6D). Consistently, the amount of LRP1 captured by DS–biotin was dramatically reduced in LRP1 knockout (LRP1^*KO*^) tumor cells generated using CRISPR-Cas9 (Fig. 5D). These results suggest that DS selectively binds to LRP1 or an LRP1-containing complex.

The direct interaction between DS and LRP1 was further validated through a cellular thermal shift assay [30]. DS treatment resulted in notable thermal stabilization of LRP1 in MC38 cells (Fig. 5E), with similar results observed in DS-treated HCT116 and E0771 cells (Fig. S6E). Additionally, LRP1 stability increased with higher DS concentrations when lysates were treated at 42 °C (Fig. 5F and Fig. S6F), and LRP1 accumulation was observed with increasing DS concentrations at a fixed enzyme–lysate ratio (Fig. 5G and Fig. S6G). Furthermore, DS-induced inhibition of lysosomal activity, evidenced by reduced DQ-BSA dequenching (Fig. 5H and Fig. S6H), decreased maturation of CTSB and CTSD (Fig. S6I), and increased yellow puncta formation (Fig. S6J), was abolished in *LRP1^KO^* HCT116 cells. Similarly, DS-induced phosphorylation of TBK1 and STAT1 was greatly attenuated in *LRP1^KO^* HCT116 cells (Fig. 5I).

LRP1 is a receptor structure as a 515-kDa extracellular α chain and an 85-kDa transmembrane β chain [31], with the α chain containing 4 ligand-binding regions (clusters I, II, III, and IV). To identify the specific LRP1 domain interacting with DS, we constructed vectors encoding truncated mutants of LRP1 (Fig. S6K). A biotin–streptavidin pull-down assay revealed that DS interacted specifically with the LRP1β domain (Fig. 5J). To confirm this interaction, we generated recombinant Flag-LRP1β protein and incubated it with increasing concentrations of DS–biotin. IB demonstrated that Flag-LRP1β was pulled down by DS–biotin in a concentration-dependent manner (Fig. 5K). Moreover, excess free DS competitively inhibited this interaction (Fig. S6L), confirming the specificity of the DS–LRP1β interaction. Surface plasmon resonance (SPR) analysis further demonstrated that DS bound to LRP1β with a dissociation constant (*K*_D_) of 9.69 μM (Fig. 5L), indicating a strong binding affinity. Importantly, DS exhibited no tumor growth-suppressive effects in mice bearing *LRP1^KO^* MC38 cells (Fig. 5M and Fig. S6M and N), highlighting the essential role of LRP1 in DS-mediated tumor growth inhibition.

To identify the specific amino acid residues in LRP1β responsible for DS binding, we performed molecular dynamics simulations using the AlphaFold-predicted LRP1β structure. Schrödinger’s docking studies identified potential binding sites, with DS forming hydrogen bond interactions with residues such as Tyr4054, Ser4134, and Asp4135 in LRP1β (Fig. 5N). Site-directed mutagenesis of these residues revealed that the D4135A mutant dramatically reduced the binding ability of LRP1β for DS (Fig. 5O and Fig. S6O). Furthermore, reintroduction of the LRP1β D4135A mutant, but not LRP1 wild type (WT), failed to restore DS-induced TBK1 and STAT1 activation in LRP1β*^KO^* cells (Fig. 5P). These findings indicate that DS directly binds to LRP1β at the Asp4135 residue.

### Combination of DS and anti-PD-1 antibody effectively suppresses tumor growth

**Fig. 6.**
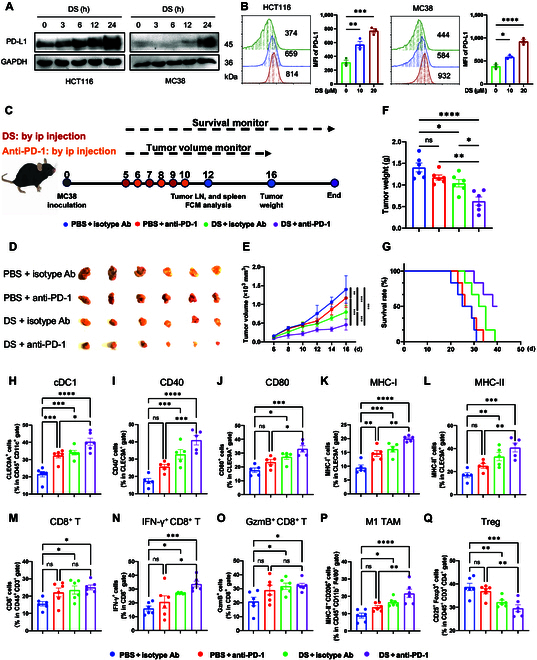
DS induces immune cell infiltration and potentiates efficacy of with anti-programmed death 1 (anti-PD-1) therapy. (A and B) HCT116 and MC38 cells were treated with DS (20 μM) for the indicated time points, the expression levels of PD-L1 were detected by immunoblotting (A), and the plasma membrane levels of PD-L1 were detected by flow cytometry analysis. Quantification of the MFI of PD-L1 is shown (B). (C to G) C57BL/6 mice bearing MC38 tumor (*n* = 6) were treated with PBS, anti-PD-1 (100 μg per mouse), DS (20 mg/kg), or their combination (C). Representative tumor images in mice are shown (D), and the tumor volume (E) and tumor weight (F) were monitored. The survival rate of mice for 40 d is shown (G). (H to Q) The proportion of cDC1s (H) and surface expression levels of CD40, CD80, MHC-I, and MHC-II (I to L) on CLEC9A^+^ cells in tumor-draining lymph nodes (TDLNs) from each group were determined by flow cytometry analysis. The proportion of CD8^+^ T cells (M), effector molecules IFN-γ^+^ cells (N) and GzmB^+^ cells (O) in CD8^+^ T-cell gates, M1 TAMs, and regulatory T cells (Tregs) in the TME from each group were determined by flow cytometry analysis. The experiments (A and B) were repeated 3 times independently with similar results. Data are presented as mean ± SEM. Statistical significance was analyzed by 1-way ANOVA test (B, F, and H to Q), or 2-way ANOVA test (E). *P < 0.05; **P < 0.01; ***P < 0.001; ns, not significant. ip, intraperitoneal; LN, lymph node; FCM, flow cytometry.

As a potent IFN-I inducer, DS markedly enhanced both the abundance (Fig. 6A and Fig. S7A) and surface expression of PD-L1 (Fig. 6B and Fig. S7B) in a concentration- and time-dependent manner across multiple tumor cell lines, including MC38, HCT116, SW620, and E0771. Consequently, we explored whether combining DS with anti-PD-1 therapy could yield synergistic anti-tumor effects. Mice bearing MC38 tumors were treated with PBS, anti-PD-1 antibody, DS, or a combination of DS and anti-PD-1 (Fig. 6C). While anti-PD-1 antibody and DS monotherapies partially reduced tumor burden compared to the control, the combination treatment resulted in the strongest suppression of tumor, as assessed in terms of tumor volume (Fig. 6D and E), tumor weight (Fig. 6F), and survival rates (Fig. 6G). Comparable efficacy of tumor growth inhibition was demonstrated in mice bearing E0771 tumors (Fig. S7C to E). To explore the immune mechanisms underlying these effects, tumors and tumor-draining lymph nodes (TDLNs) were collected 2 d following the final treatment, and the population and activation markers of DCs were analyzed by flow cytometry. The combination treatment markedly increased the population of cDC1s (CLEC9A^+^ CD11c^+^) in TDLNs but not within tumors (Fig. 6H and Fig. S7F), suggesting that DCs may migrate from tumors to TDLNs, where they potentially activate T cells. Furthermore, the combination treatment up-regulated the expression of costimulatory molecules (CD40 and CD80) and MHC-I on cDC1s in both TDLNs and tumors (Fig. 6I to K and Fig. S7G). Notably, MHC-II expression was markedly elevated in the TDLNs but not in the tumors of the combination treatment group (Fig. 6L and Fig. S7G). These observations suggest that the combination treatment effectively triggered the maturation of DCs. Analysis of the TME revealed that the combination regimen augmented the proportion of tumor-infiltrating CD8^+^ T cells compared to monotherapy (Fig. 6M). Additionally, the production of effector molecules by tumor-infiltrating CD8^+^ T cells, including IFN-γ and GzmB, was remarkably increased following DS and anti-PD-1 antibody combination treatment (Fig. 6N and O), further supporting the activation of cDC1s and cytotoxic CD8^+^ T cells. Flow cytometric analysis also showed that macrophages were polarized toward the M1 phenotype in the combination therapy group (Fig. 6P). Conversely, the populations of immunosuppressive regulatory T cells (Tregs) and monocytic myeloid-derived suppressor cells (M-MDSCs) were substantially reduced within the TME in response to the combination treatment (Fig. 6Q and Fig. S7H and I). While the overall population of NKT cells in the tumors remained unchanged, the percentage of activated NKT cells (IFN-γ^+^) increased in response to the combination treatment (Fig. S7J to L). These findings suggest that DS potentiates the anti-tumor effects of anti-PD-1 therapy by reprogramming the immune microenvironment.

### DS enhances anti-tumor efficacy in combination with a STING agonist

**Fig. 7.**
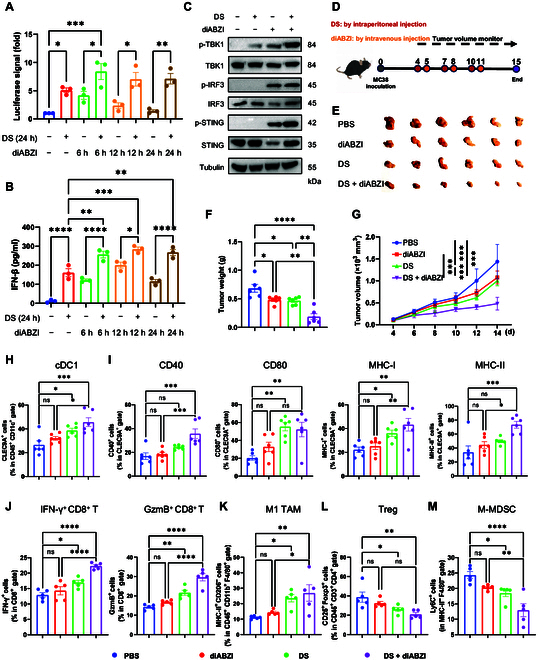
DS enhances the anti-tumor effects when combined with a stimulator of interferon genes (STING) agonist. (A) MC38 cells were treated with DMSO, DS (20 μM) for 24 h, and/or diABZI (100 nM) for 0, 6, 12, and 24 h. The activity of the IFN-β reporter gene was subsequently assessed. (B) MC38 cells were treated with DMSO or DS (20 μM) for 24 h, as well as diABZI (100 nM) for 0, 6, 12, and 24 h. The levels of IFN-β in the supernatant were analyzed by ELISA. (C) Immunoblotting analysis of the phosphorylation levels of STING, TBK1, and IRF3 in MC38 cells treated with DMSO, DS (20 μM), and/or diABZI (100 nM) for 12 h. (D to M) C57BL/6 mice bearing MC38 tumor (*n* = 6) were treated with PBS, diABZI (100 μg/kg), DS (20 mg/kg), or a combination of both treatments (D). Representative tumor images in mice are shown (E), and the tumor volume (F) and tumor weight (G) were monitored. The proportions of cDC1 (H) and surface expression levels of CD40, CD80, MHC-I, and MHC-II on CLEC9A^+^ cells (I) in the TME from each group were determined by flow cytometry analysis. The proportions of IFN-γ^+^ cells and GzmB^+^ cells in CD8^+^ T-cell gates (E), M1 TAMs (K), Tregs (L), and monocytic myeloid-derived suppressor cells (M-MDSCs) (M) in the TME from each group were determined by flow cytometry analysis. The experiments (A and B) were repeated 3 times independently with similar results. Data are presented as mean ± SEM. Statistical significance was analyzed by 1-way ANOVA test (A, B, F, and H to M), or 2-way ANOVA test (G). *P < 0.05; **P < 0.01; ***P < 0.001; ns, not significant.

It is well established that a STING agonist activates the downstream IFN-I signaling pathway in tumor cells [22]. Given our observation that DS induces a TBK1-dependent but STING-independent activation of IFN-I, we hypothesized that combining DS with a STING agonist could enhance IFN-I signaling in tumor cells, potentially resulting in a synergistic anti-tumor effect. Previous studies have shown that STING activation occurs rapidly but is subsequently down-regulated due to lysosomal degradation of STING, and inhibiting this degradation enhances IFN-I signaling and the anti-tumor response [32,33]. To evaluate the effects of DS and a STING agonist on IFN-I signaling, we treated MC38 and HCT116 cells with DS, diABZI (a specific STING agonist) [34], or a combination of both. IFN-β reporter assay and ELISA confirmed that the combination treatment synergistically enhanced IFN-I pathway activation at 6 and 12 h post-diABZI treatment (Fig. 7A and B). By 24 h, while IFN-I activation induced by diABZI monotherapy diminished, the combination treatment sustained the IFN-I pathway activation (Fig. 7A and B). IB analysis further revealed that diABZI treatment for 12 h activated STING and TBK1 but also led to STING degradation (Fig. 7C). In contrast, the combination treatment enhanced the phosphorylation of TBK1 and IRF3 while reducing STING degradation (Fig. 7C), indicating DS both trigger IFN-I responses independently and attenuates STING degradation, thereby sustaining IFN-I signaling. To validate these findings in vivo, we used the MC38 tumor model, treating mice with DS, a low dose of diABZI (100 μg/kg), or their combination (Fig. 7D). The combination treatment resulted in a more pronounced and synergistic suppression of tumor growth and tumor weight compared to either treatment alone (Fig. 7E to G). To investigate the underlying mechanisms, we analyzed the tumor-infiltrating immune cells and their activation markers using flow cytometry. The combination treatment markedly expanded the population of cDC1s relative to the monotherapy groups (Fig. 7H). Furthermore, cDC1s exhibited substantially elevated the expression of costimulatory markers (CD40 and CD80) and antigen-presenting molecules (MHC-I and MHC-II) following the combination treatment group (Fig. 7I), suggesting that DS enhances the infiltration and maturation of DCs within the TME. Additionally, the combination therapy additionally enhanced CD8^+^ T-cell effector function, evidenced by elevated IFN-γ secretion and GzmB production (Fig. 7J). The population of tumor-associated M1 macrophages was also markedly increased (Fig. 7K), while the populations of immunosuppressive Tregs and M-MDSCs were reduced compared to those in the PBS and diABZI groups (Fig. 7L and M). These findings collectively demonstrate that the combination of DS and diABZI effectively reprograms the immune microenvironment, thus enhancing anti-tumor immunity.

## Discussion

**Fig. 8.**
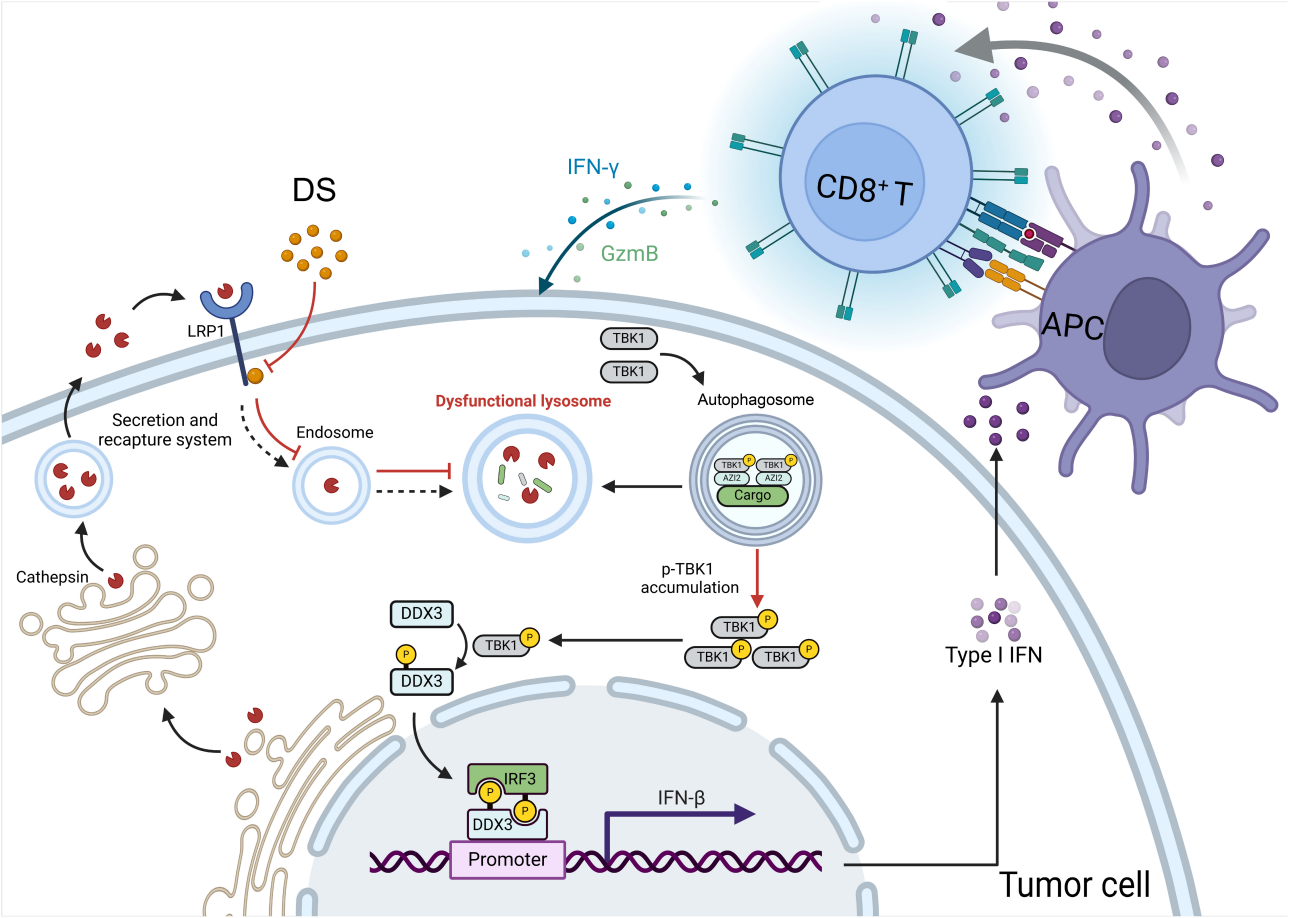
Proposed model of DS enhancing anti-tumor immunity through activating type I IFN signaling. DS directly interacts with LRP1, thereby inhibiting the lysosomal secretion–recapture system, which results in lysosomal dysfunction. This dysfunction leads to the accumulation of p62 and the adaptor protein AZI2, which mediates the activation of TBK1. Consequently, this process enhances type I IFN signaling and promotes anti-tumor immunity. The figure was generated by BioRender.com. APC, antigen-presenting cell.

As endogenous anti-tumor immunity is often compromised during tumor development [35], there is an urgent need for exogenous therapeutic strategies to enhance tumor treatment. IFN-I pathway activation has been positively correlated with improved clinical anti-tumor immune responses, and intra-tumor IFN-I levels have served as a predictive biomarker for the clinical outcomes of cancer patients [36]. In this study, utilizing an IFN-β-Luc reporter screening approach, we identified DS as a potent IFN-I inducer capable of promoting anti-tumor adaptive immunity. We demonstrated, for the first time, that DS markedly inhibits tumor growth and sensitizes tumors to PD-1 blockade by activating TBK1-dependent IFN-I signaling through targeting LRP1 (Fig. 8). These findings highlight the potential of DS to remodel immunologically “cold” tumors into “hot” tumors, thereby enhancing their responsiveness to immunotherapy.

Previous studies have shown that DS inhibits tumor angiogenesis, epithelial–mesenchymal transition, and proliferation and modulates autophagy in various human cancer cell lines [37–39]. Here, we revealed that the anti-tumor activity of DS is mediated through TBK1-dependent IFN-I signaling. Our findings demonstrate that DS treatment robustly activates IFN-I pathways in tumor cells, enhancing tumor immunogenicity by facilitating antigen presentation and reprogramming the TME by recruitment and activation of DCs and T cells. Importantly, we found that DS-induced IFN-I activation in tumor cells is dependent on TBK1; silencing TBK1, but not MAVS, STING, or MyD88, abolished DS-mediated IFN-I activation. This conclusion is supported by recent evidence demonstrating that TBK1 promotes intratumoral CD8^+^T cell infiltration and enhances tumor sensitivity to ICB therapy [40]. Together, these findings highlight the critical involvement of tumor-intrinsic, TBK1-dependent IFN-I signaling in mediating the anti-tumor effect of DS.

We provided evidence that DS exerts its anti-tumor activity by remodeling the tumor immune microenvironment. Accumulating studies suggest that reactivating DCs, CD8^+^ T cells, and macrophages in the TME represents an effective therapeutic strategy against tumors. Our in vitro and in vivo results demonstrated that DS promotes DC maturation and enhances the secretion of effector molecules (IFN-γ/GzmB) by tumor-infiltrating CD8^+^ T cells, establishing the role of DS in orchestrating DC-CD8^+^ T-cell immunity for tumor suppression. This aligns with previous reports showing that IFN-I promotes DC maturation, cross-presentation capacity, and CD8^+^ T-cell expansion [6]. M1 macrophages exert anti-tumor activity by secreting pro-inflammatory factors and engaging in phagocytosis, whereas M2 macrophages support tumor progression [41]. We showed that DS reprograms macrophages toward an M1 phenotype, enhancing their phagocytic activity and contributing to anti-tumor effects. Previous study suggests that IFN-I released by tumor cells can act directly on NKT cells [42]. Consistently, we demonstrated that DS increases NKT cell infiltration, thus promoting anti-tumor immunity. Collectively, these findings provide clear evidence that DS suppresses tumor growth by orchestrating comprehensive immune reprogramming within the TME. As a natural compound, DS may exhibit off-target effects; thus, using low doses of DS can minimize these while preserving its anti-tumor and immunomodulatory effects. Additionally, advanced formulations (e.g., liposomes, nanoparticles, or microemulsions) may enhance DS’s therapeutic efficacy and clinical applicability.

A key finding of this study is that DS triggers LRP1-mediated TBK1 activation. LRP1 primary functions by ligand binding, ligand endocytosis, lysosomal delivery, and receptor recycling back to the cell membrane [43]. Aberrant overexpression of LRP1 has been observed in the malignancy and driven progression in pancreatic, breast, and endometrial cancers [44–46]. LRP1 also drives tumorigenesis and tumor progression by activating AKT/mTOR signaling [47] and inhibiting JNK and NF-κB pathways [48]. However, DS treatment did not affect the phosphorylation levels of NF-κB, Akt, mTOR, or their downstream targets (data not shown). LRP1 is known to facilitate the targeting of lysosomal proteins like CTSD and CTSB to the lysosomal compartment [49,50]. Consistently, our results demonstrate that DS specifically binds to LRP1, impairing lysosomal function and inducing AZI2-dependent TBK1 activation. Thus, LRP1 represents a promising target for activating IFN-I signaling, which may facilitate the development of potent immunotherapies.

The development of combination therapies to enhance ICB efficacy is a promising frontier in cancer immunotherapy. Clinical trials are currently exploring strategies combining IFN-I inducers with ICB monotherapy. Our work demonstrates that combination treatment with DS plus either anti-PD-1 or diABZI substantially remodels the tumor immune microenvironment. This combinatorial approach promotes CD103^+^ DC maturation, facilitates T-cell activation and expansion, and induces M2-to-M1 macrophage polarization, ultimately enhancing cytotoxic CD8^+^ T-cell infiltration. These findings are consistent with established correlations between cDC1-dependent T-cell infiltration [51], M1 macrophage prevalence [52], and improved clinical outcomes. Notably, our combination therapy effectively counteracts the immunosuppressive TME by reducing M-MDSC and Treg infiltration, which are known to promote tumor progression through secreting inhibitory cytokines (IL-6, IL-10, and TGF-β) [53,54]. The combination of DS with the STING agonist diABZI demonstrates enhanced anti-tumor efficacy via complementary mechanisms: (a) DS activates IFN-I signaling through a STING-independent pathway, while diABZI engages the canonical STING–IFN axis, generating dual signaling cascades that synergistically amplify IFN-I production; (b) DS inhibits lysosome-mediated STING degradation, thereby extending diABZI-driven STING activation; and (c) sustained IFN-I signaling enhances DCs-dependent T-cell priming while suppressing immunosuppressive cell populations, including Tregs and M-MDSCs. This multi-level synergy overcomes the transient efficacy limitation of STING monotherapy, offering a rational combination strategy for immunotherapy-resistant tumors.

In summary, our findings demonstrate that DS elicits anti-tumor immunity by targeting LRP1 and engaging DCs, T cells, and macrophages. DS specifically binds to LRP1, disrupts lysosomal function, and activates TBK1-dependent IFN-I signaling, thereby reprogramming the TME and enhancing the tumor’s response to anti-PD-1 or STING agonist treatment. These findings offer a compelling foundation supporting the potential application of DS as a promising and effective immune modulator to improve the efficacy of current ICB therapies.

## Materials and Methods

### Cell lines, chemicals, and antibodies

Human colorectal cancer cell lines HCT116 and SW620, mouse colorectal cancer cell line MC38, and mouse breast cancer cell line E0771, as well as 293T, NIH 3T3, and DC2.4 cell lines, were procured from the Institute of Basic Medicine, Chinese Academy of Medical Sciences (Beijing, China). MC38-OVA cells were generated through stable transfection of MC38 cells with the plasmid pCI-neo-mOVA (Addgene, #25099). The B3Z mouse hybridoma cell line was generously provided by Dr. Nilabh Shastri (University of California, Berkeley, CA, USA). The cell lines MC38, DC2.4, and B3Z were cultured in RPMI 1640 medium, while HCT116, SW620, E0771, and NIH 3T3 were grown in Dulbecco’s modified Eagle medium. Both media were supplemented with 10% fetal bovine serum (HyClone, Logan, UT, USA) and 100 U/ml penicillin–streptomycin, and cells were maintained at 37 °C in a humidified 5% CO_2_ atmosphere.

Bone marrow (BM) cells were harvested from the femurs and tibias of 6- to 8-week-old female C57BL/6J mice through mechanical flushing and subsequent passage through a 70-μm cell strainer. Following centrifugation, erythrocytes were lysed using red blood cell lysis buffer (150 mM NH_4_Cl, 10 mM KHCO_3_, and 0.1 mM Na_2_EDTA) for 3 min. The purified BM cells were then maintained in complete RPMI 1640 medium, supplemented with either: (a) 50 ng/ml mGM-CSF and 20 ng/ml mIL-4 (PeproTech) for BMDC differentiation, or (b) 20 ng/ml mM-CSF (PeproTech) for BMDM generation. The medium was refreshed every 3 d. After 7 d, over 90% of the BM cells had differentiated into BMDCs or BMDMs.

Chemical monomers of natural products were obtained from MedChemExpress (Shanghai, China). Additional experimental reagents and commercial kits utilized in this work are compiled in Table S1, with antibody details presented in Tables S2 and S3.

### Xenograft tumor models and treatment

Female C57BL/6J mice and BALB/c nude mice (6 to 8 weeks old) were commercially sourced from HFK Bio-Technology Co., Ltd (Beijing, China) and maintained under specific-pathogen-free conditions. All animal experiments were conducted in accordance with the Institute of Medicinal Biotechnology (IMB) animal ethics committee, Chinese Academy of Medical Sciences (approval numbers: IMB-20230830D202, IMB-20230922D202, IMB-20231030D202, IMB-20240103D6, IMB-20240611D202, and IMB-20240927D208). For tumor models, MC38 (1 × 10^6^) or E0771 (2 × 10^6^) cells were subcutaneously injected into the right flank of respective mouse strains. DS or vehicle was administered intraperitoneally (10, 20, or 40 mg/kg) at designated intervals. Anti-PD-1 antibody (100 μg/mouse) was delivered intraperitoneally every other day for a total of 3 doses. For DC depletion, 5 mg of Cyt C (Sigma, #C2506) or vehicle was injected intravenously on days −1, 7, and 14. Macrophage depletion was achieved using clodronate liposomes (100 μl; FormuMax Scientific Inc., Sunnyvale, CA, USA) or control liposomes intraperitoneally on days −1, 5, and 11. Anti-CD8α and isotype control antibodies were administered intraperitoneally (100 μg per mouse) on days −1, 4, and 8. Body weight and tumor growth were measured every 48 h, with tumor volume calculated as *π*/6 × tumor length × (tumor width) [2]. Terminal tumor tissues were collected for tumor-infiltrating immune cells analysis by flow cytometry.

### IFN-β reporter assay

The day prior to transfection, 5 × 10^4^ HCT116 cells were seeded into 24-well plates to achieve 80% to 90% confluency at the time of transfection. After replacing the medium with serum-free medium 1 h pretransfection, cells were cotransfected with the IFN-β-pGL3 plasmid (Addgene, #102597) and pRL-TK plasmid (Beyotime, D2760) using the Lipofectamine 2000 transfection reagent, at plasmid amounts of 100 and 10 ng per well. Twelve hours posttransfection, the medium was replaced with a fresh complete medium containing either PBS or the compound, followed by incubation for 24 h. Luciferase expression was measured using the Dual-Luciferase Reporter Assay System (Promega). Briefly, cells were lysed in 100 μl of Passive Lysis Buffer (15- to 20-min shaking), and 10 μl of the lysate was combined with 50 μl of Luciferase Assay Substrate for initial reading. After adding 50 μl of Stop & Glo Buffer, the plate was remeasured for *Renilla* activity. The ratio of luciferase to *Renilla* values indicated IFN-β transcriptional activity.

### Quantitative real-time PCR

Total RNA was isolated with TRIzol Universal (Thermo Fisher Scientific) following the manufacturer’s instructions. Complementary DNA synthesis and quantitative PCR were performed using the UniPeak One Step RT-qPCR SYBR Green Kit (Vazyme Biotechnology, R226). Relative messenger RNA expression levels were calculated by the ΔΔCt method with glyceraldehyde-3-phosphate dehydrogenase as endogenous control. All primer sequences, designed via Primer3Plus, are provided in Table S4.

### ELISA and IB

Levels of IFN-β and CXCL10 in cell culture supernatants were measured by ELISA kits (R&D Systems, DIFNB0, MIFNB0, DIP100, and DY466-05) as directed by the manufacturer. IB was performed as described before [30].

### Cell viability assay

For 3-(4,5-di methyl thiazol-2-yl)-2,5-diphenyltetrazolium bromide (MTT) assays, cells in 96-well plates were treated with DS (various concentrations, 48 h, 37 °C, 5% CO_2_), followed by 4-h incubation with MTT solution (5 mg/ml, 20 μl/well). The supernatant was then discarded, and the formazan crystals were dissolved in DMSO (100 μl/well) shaken for 20 min. Absorbance was quantified at 570 nm. For crystal violet assay, DS-treated cells in 6-well plates were fixed and stained with 0.3% crystal violet (10 min, room temperature) after colony development. Following 3 washes, images of the stained colonies were captured.

### Measurement of DC activation, T-cell activation, and macrophage polarization [30]

To assess DC activation and antigen presentation, we treated MC38-OVA cells with DS for 24 h in 6-well plates, followed by coculture with BMDCs or DC2.4 for another 24 h. Harvested cells were stained with fluorescent antibodies targeting CD11c, CD40, CD80, CD86, MHC-I, MHC-II, and H2Kb-SIINFEKL.

For T-cell activation analysis, B3Z cells were seeded at a 5:1 ratio onto the previously cocultured BMDCs or DC2.4 and further coincubated for 16 h. Then, harvested cells were stained with fluorescent antibodies against CD8α, CD69, IFN-γ, and GzmB.

To evaluate BMDMs’ phagocytic capacity, MC38 cells were prestained with carboxyfluorescein succinimidyl ester (CFSE) for 10 min and then either treated or not treated with DS. Following a 24-h incubation in DS-containing medium, the cells were coincubated with BMDMs for an additional 24 h. Harvested cells were then stained using CD45, CD11b, F4/80, and CFSE fluorescent antibodies. For macrophage polarization assays, harvested cells were stained using CD45, CD11b, F4/80, CD206, and MHC-II fluorescent antibodies. Flow cytometry was performed on a Guava easyCyte system, with data analyzed using guavaSoft 3.1.

### CRISPR/Cas9-mediated knockout [55]

Single guide RNAs were designed via the CHOPCHOP online tool (http://chopchop.cbu.uib.no/) and subsequently cloned into the LentiCRISPRv2 vector. When 293T cells reached approximately 80% confluency, they were transfected with a total of 11 μg of lentiviral packaging plasmid (5 μg of LentiCRISPRv2, 5 μg of psPAX2, and 1 μg of VSVG) using Lipofectamine 2000. Supernatants were collected at 72 h posttransfection, filtered using a 0.45-μm filter, and used to transduce HCT116 or MC38 cells for 24 h before puromycin selection. The single guide RNA sequences are listed in Table S5.

### RNA sequencing

Total RNA was isolated from DS- or DMSO-treated MC38 cells using TRNzol Universal Reagent according to the manufacturer protocols. RNA sequencing was performed by OE Biotech Co., Ltd. (Shanghai, China) on the HiSeq X10 platform. Raw sequencing data were quality-filtered using Trimmomatic and aligned to the mm10/GRCm38 mouse genome with HISAT.

### Autophagy assay

To differentiate autophagosomes from autolysosomes, HCT116 cells were transfected with the mCherry-EGFP-LC3 plasmid using Lipofectamine 2000. Following 24-h expression and subsequent treatment with PBS or DS, cells were imaged by confocal microscopy (Olympus).

### Lysosomal function assay

WT and LRP1*^KO^* HCT116 cells were treated with 20 μM DS or DMSO for 12 h. To evaluate lysosomal proteolysis, cells were stained with DQ-Red BSA (10 μg/ml) (Molecular Probes/Invitrogen, D-12051) for 6 h, washed, and visualized via confocal microscopy (Olympus). Red fluorescence was quantified by flow cytometry under green laser excitation (RED-G). For LysoSensor staining, HCT116 cells were incubated in media containing 20 μM DS or 200 nM BafA1 for indicated durations. Then, cells were stained with 1 μM LysoSensor DND-189 (Invitrogen) for 10 min (37 °C, 5% CO_2_). After 3 PBS washes, cells were visualized using fluorescence microscopy (Zeiss, Axio Vert A1).

### Cell fractionation assay

Lysosomal fractions were purified from HCT116 and MC38 cell homogenates following the Sigma-Aldrich LYSISO1 kit protocol. Sequential centrifugation was performed (1,000 × *g* for 10 min followed by 20,000 × *g* for 20 min, both at 4 °C) to obtain crude lysosomal pellets. The 20,000 × *g* supernatant was retained as the cytosolic fraction, while the pellet, representing the lysosomal fraction, was solubilized in the specified lysis buffer according to the protocol prior to immunoblot analysis.

### Immunofluorescence

Cells were seeded onto 8-well chamber slides (Thermo Fisher Scientific) for 24 h. After being washed, cells were fixed with absolute ethanol for 15 min at room temperature. After permeabilization with 0.5% Triton X-100 and blocking with 5% BSA at room temperature for 1 h, samples were incubated with primary antibodies overnight at 4 °C, followed by species-matched secondary antibodies for 1 h at room temperature. Nuclei were stained using 4′,6-diamidino-2-phenylindole (Cell Signaling Technology), and images were acquired using an Olympus confocal microscope. Fluorescence intensity and colocalization were analyzed with ImageJ v1.53e.

### Biotin–DS pull-down assay

Streptavidin–agarose beads and biotin–DS were preincubated with HEPES buffer (20 mM HEPES, pH 7.4, 1 mM dithiothreitol, 100 mM NaCl, and 0.05% NP-40) at 25 °C for 2 h. Lysates from HCT116, MC38-WT, MC38-LRP1*^KO^*, or 293T cells expressing LRP1 fragment plasmids were preincubated with varying concentrations of DS or DMSO at 37 °C with 5% CO_2_ for 2 h. Then, these lysates were incubated with the bead complex overnight at 4 °C. After 3 HEPES buffer washes, proteins bound to the beads were eluted and analyzed by IB using anti-Flag antibody or anti-LRP1 antibody.

### Site-directed mutagenesis

LRP1 point mutants were constructed via Fast Site-Directed Mutagenesis Kit (Tiangen Biotech, KM101) and sequence-confirmed by Tsingke Biotech (Beijing, China). Streptavidin–agarose beads were incubated with DS–biotin in HEPES buffer (2 h, 25 °C). 293T cell lysates expressing Flag-LRP1-WT and Flag-LRP1-S4143A, -D4135A, -Y4054A, -K4154R, -G4166A, and -P4265A were preincubated with DS or vehicle control (2 h, 37 °C, 5% CO_2_) prior to overnight pull-down at 4 °C. Following 3 HEPES buffer washes, bead-bound proteins were analyzed by IB with anti-Flag or anti-LRP1 antibodies.

### Cellular thermal shift assay [30]

MC38 or HCT116 cells (2 × 10^7^) were digested, resuspended in PBS, and treated with DMSO or 50 μM DS at 37 °C for 1 h. Cell suspension was then aliquoted into 8 PCR tubes (100 μl per tube). PCR tubes were heated for 3 min at designated temperatures (33 to 51 °C) in a 96-well thermal cycler. After 3-cycle room-temperature cooling for 3 min and liquid nitrogen flash freezing, samples were centrifuged at 20,000 × *g* for 20 min at 4 °C. The supernatant was denatured with 5× loading buffer and heated at 100 °C for 10 min. The prepared samples were analyzed by IB. All experiments were conducted in triplicate.

### Drug affinity responsive target stability assay

MC38 cells (2 × 10^7^) were lysed in ice-cold buffer with protease/phosphatase inhibitors for 30 min, with occasional gentle shaking. After centrifugation at 12,000 rpm for 15 min, the supernatant was collected, and protein concentrations were quantified via the Bradford assay. Protein lysates were then incubated with either DMSO or DS (50 μM) at 37 °C for 1 h. The Pronase (Sigma-Aldrich, #107433) volume was calculated based on the protein concentration (Pronase-to-protein mass ratio = 1:2,500) and added for 30 min at 37 °C before terminating reactions with 5× loading buffer. Afterward, samples were heated at 100 °C for 10 min. The processed samples were analyzed by IB in triplicate.

### SPR analysis

The SPR measurements were performed by XLEMENT WeSPR One Biomolecular Interaction Analyzer at room temperature. Recombinant human His-LRP1β was immobilized onto an anti-His tag sensor chip. DS was dissolved in 5% DMSO in PBS with Tween. The system was perfused with DS solutions at the indicated concentrations (40 μl/min flow rate, 150-s duration). Binding parameters were analyzed using the SPRAutolink software.

### Molecular docking

The crystal structure of the LRP1 domain was predicted using the AlphaFold 3 algorithm (https://alphafoldserver.com/). The target protein (LRP1) was prepared using the protein preparation wizard tool (Schrödinger Suite, 2018), which involved removing extraneous chains and water molecules, as well as optimizing the orientations of hetero groups in the PDB structure. The ligand structures were prepared using Schrödinger’s LigPrep module, which generates low-energy conformers; accounts for tautomers, ionization states, stereochemistry; and ensures correct chirality. The ligand was then minimized using the OPLS 2005 force field. For molecular docking, pull-down experiments using a biotin-labeled ligand were performed to identify the key binding regions of LRP1 (LRP1β). A docking study was carried out with the Maestro module of Schrödinger’s docking suite to predict ligand-binding interactions with LRP1β. Glide XP was used for docking calculations with the OPLS 2005 force field to estimate binding affinities between LRP1β and DS.

### Preparation and flow cytometric analysis of tumor-infiltrating immune cells [30]

Tumor tissues were collected, mechanically dissociated, and enzymatically digested in a medium containing collagenase and Cell Stimulation Cocktail (phorbol 12-myristate 13-acetate/ionomycin with brefeldin A) on a shaker at 37 °C, 180 rpm, for 30 min. The digested tissue was then filtered through 70-μm mesh. Single-cell suspensions were stained with fluorochrome-conjugated antibodies against T-cell markers (CD45, CD3, CD8, IFN-γ, GzmB, Ki67, and NK1.1), DC markers (CD45, CD11c, CD103, CLEC9A, MHC-II, CD80, CD86, and CD40), macrophage polarization markers (CD45, CD11b, F4/80, CD86, MHC-II, and CD206), Treg markers (CD45, CD3, CD4, CD25, Foxp3, Ki67), and MDSC subsets (CD45, CD11b, MHC-II, F4/80, Ly6C, and Ly6G). Flow cytometry was performed with the Guava easyCyte platform, with data analyzed using the guavaSoft 3.1 software.

### Statistics

Data are presented as mean ± standard error of mean. Statistical analyses were performed using a 2-tailed unpaired Student *t* test for direct comparisons between 2 experimental groups. For experiments involving multiple comparisons, 1-way or 2-way analysis of variance was performed followed by Bonferroni’s post hoc test. All graphical representations and statistical computations were generated by GraphPad Prism 10, and *P* values <0.05 were considered statistically significant.

## Data Availability

The data that support the findings of this study are available from the corresponding authors upon reasonable request.
