# Supplementary material for: Daurisoline Modulates the TBK1-Dependent Type I Interferon Pathway to Boost Anti-tumor Immunity via Targeting of LRP1
Source: Research (Wash D C). 2025 Jul 4;8:0764. doi: 10.34133/research.0764 (PMC12231193; doi:10.34133/research.0764)
Supplement: Supplementary 1 — Figs. S1 to S8 Tables S1 to S6 [file research.0764.f1.docx]

**Daurisoline modulates the TBK1-dependent type Ⅰ interferon pathway to boost anti-tumor immunity via targeting of LRP1**

Borui Tang^1†^, Yuting Wang^2†^, Liping Li^2†^, Cuicui Sun^2†^, Jingwen Dong^2^, Ruoqi Li^2^, Jianfeng Wang^3^, Yu Long^2^, Mingxiao Yin^4^, Fei Xie^3^, Dian Xiao^3^, Xinbo Zhou^3^, Na Zhang^2*^, Xiuli Zhao^1*^, Yanchun Feng^5*^, Hongbin Deng^2^*^*^*

**This file includes:**

**Fig S1. related to Fig 1**. DS is a potent IFN-β inducer.

**Fig S2. related to Fig 2**. DS-treated tumor cells promote DCs maturation, macrophage polarization and T cell activation.

**Fig S3. related to Fig 3**. In vivo evaluation of the safety of DS.

**Fig S4. related to Fig 3**. DS inhibits tumor growth and stimulates antitumor immunity.

**Fig S5.** **related to Fig 4**. DS-mediated lysosomal inhibition induces IFN-I activation.

**Fig S6. related to Fig 5**. LRP1 is the direct target protein of DS.

**Fig S7. related to Fig 6**. DS induces immune cell infiltration and potentiates efficacy of anti-PD-1 therapy.

**Fig S8**. Gating strategies for flow cytometry analysis.

**Table S1**. Reagents and commercial assay kits.

**Table S2**. Antibodies used for immunoblotting.

**Table S3**. Antibodies used for flow cytometry, immunofluorescence and IHC assays.

**Table S4**. Forward and reverse primers for qPCR.

**Table S5**. sgRNA sequence for knocking out the indicated proteins.

**Table S6**. Plasmids used in this study.

**Fig S1****. DS is a potent IFN-β inducer.** (A) HCT116 and MC38 cells were treated with DS (10, 20, 30, and 40 μM) for 24 hours, cell viability was determined with a CCK-8 kit. (B) MTT assay were used to examine the effect of DS on the viability of DC2.4, NIH3T3, ANA-1, HCT116 and MC38 cells. (C, D) MC38 (C) or SW620 (D) cells were treated with indicated concentrations of DS for 24 hours or treated with 20 μM DS for indicated time points, the phosphorylation levels of TBK1 and STAT1 were determined by immunoblotting. Data were shown as mean ± SEM of 3 independent experiments. ^*^*p <* 0.05, ^***^*p <* 0.001, ns, not significant.
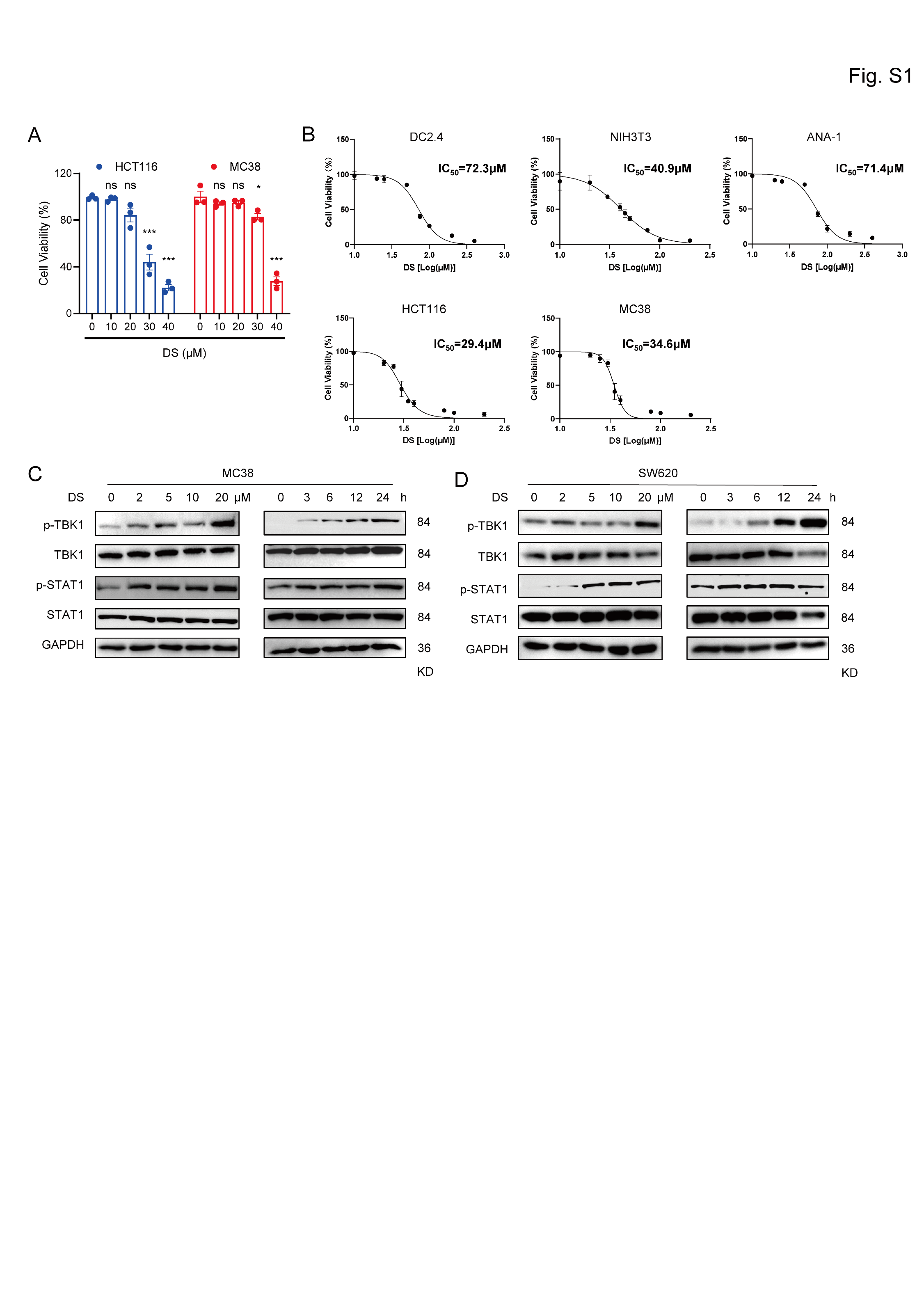


**
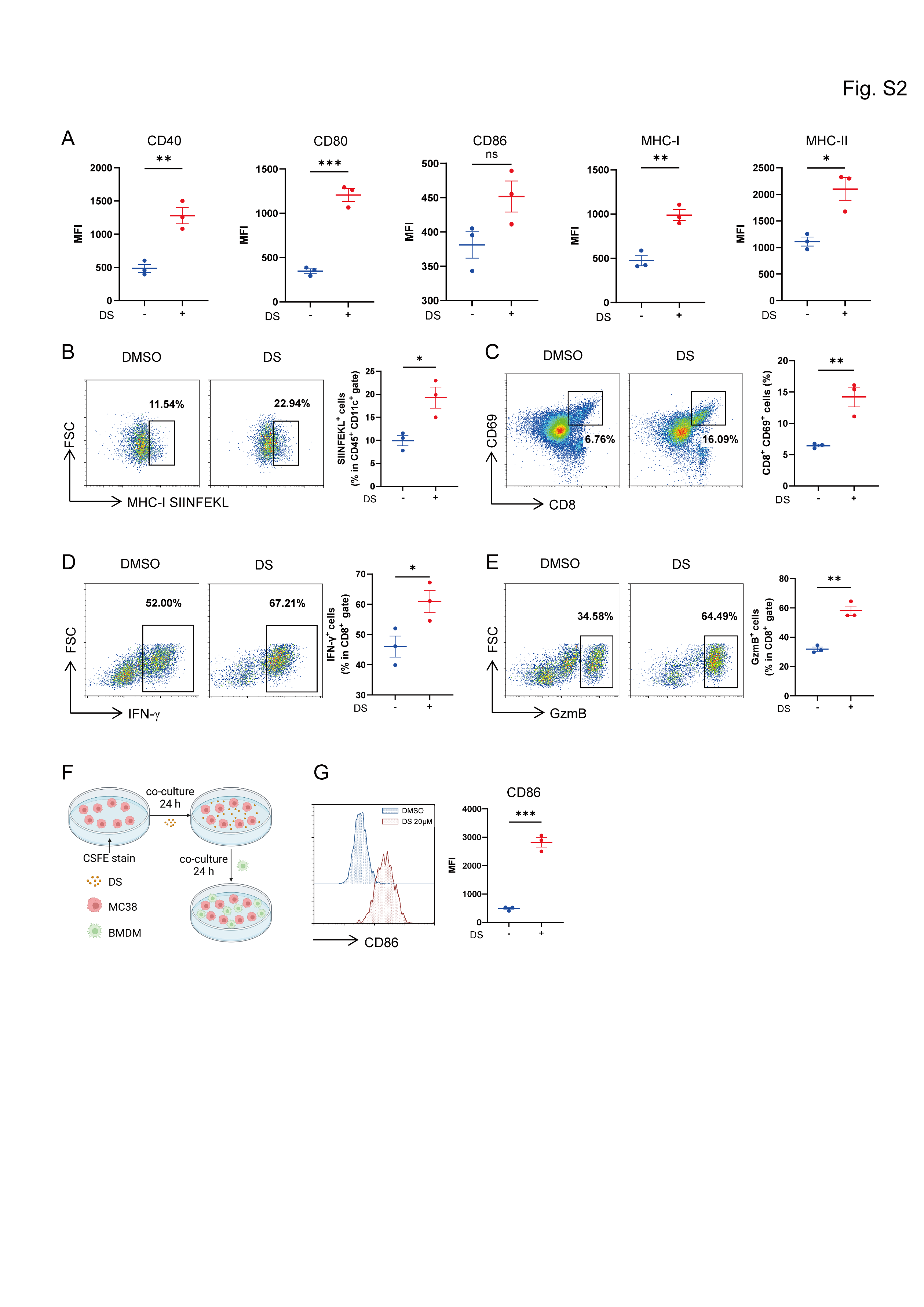
Fig S2****. DS-treated tumor cells promote DCs maturation, macrophage polarization and T cell activation.** (A, B) MC38-OVA cells were treated with DMSO or DS (20 μM) for 24 hours, then cocultured with DC2.4 for an additional 18 hours, after which, surface expressions of CD40, CD80, CD86, MHC-I, MHC-II (A), and MHC-I-SIINFEKL (B) on DC2.4 were determined by flow cytometry analysis. (C-E) MC38-OVA cells were treated with DMSO or DS (20 μM) for 24 hours, followed by coculturing with DC2.4for 24 h and B3Z cells for an additional 16 hours, then the activation of B3Z cells was measured by surface expression of CD69 (C), effector molecules IFN-γ (D), and GzmB (E) production. (F) The diagram of CFSE stained MC38 coculturing with BMDMs. (G) MC38 cells were treated with DMSO or DS (20 μM) for 24 hours, then cocultured with BMDMs cells for an additional 24 hours, after which, surface expression of CD86 on BMDMs were monitored by flow cytometry analysis. Data were presented as mean ± SEM. ^*^*p* < 0.05, ^**^*p* < 0.01, ^***^*p* < 0.001, ns, not significant.

**
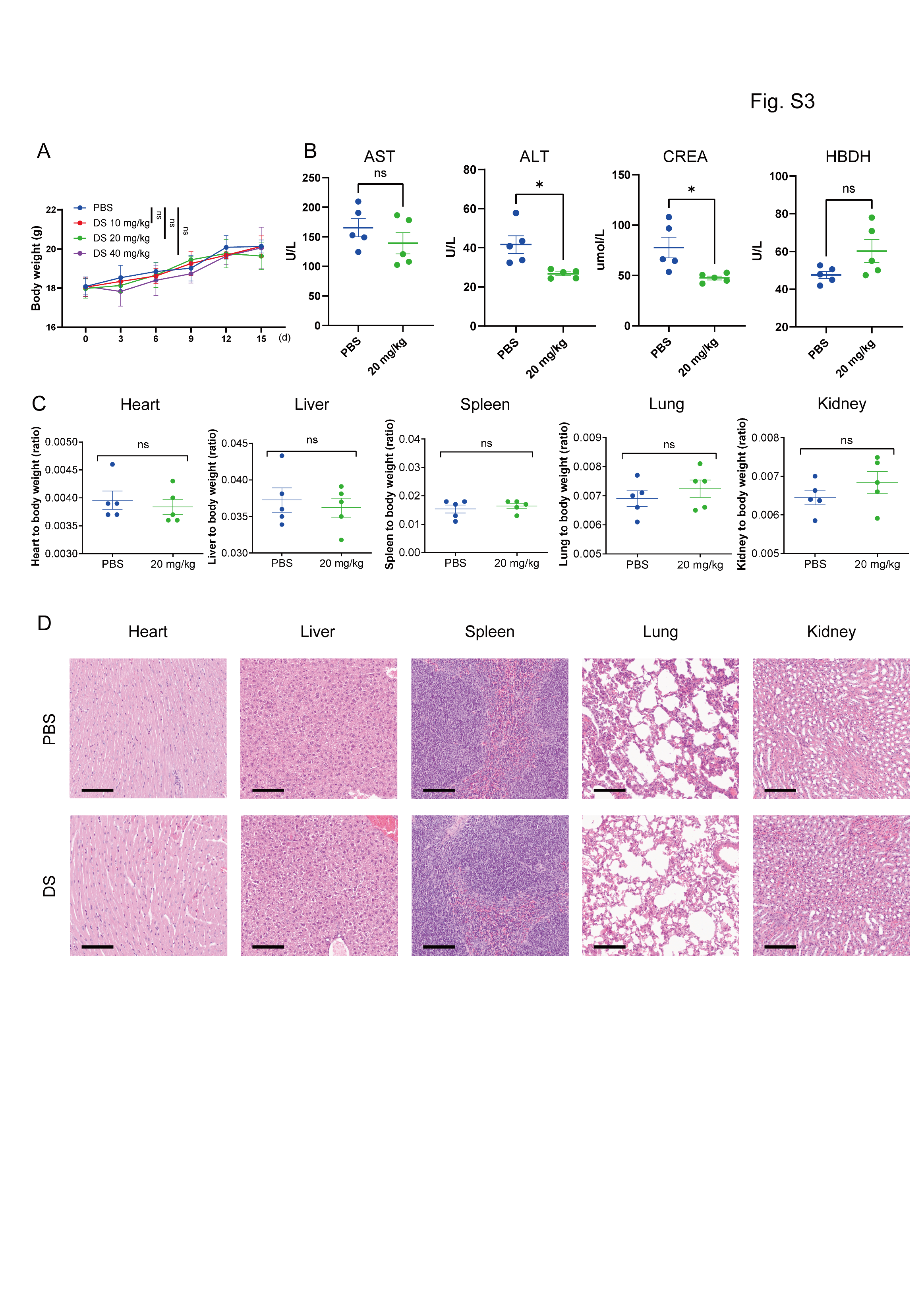
Fig S3****.** **In vivo evaluation of the safety of DS.** (A) C57BL/6 mice with subcutaneous MC38 tumor (n=5) were *i.p.* injected with PBS or DS (10, 20, and 40 mg/kg), the body weights of PBS and 40 mg/kg group were monitored for 16 days. (B) Serum biochemistry parameters, including AST, ALT, CREA and HBDH were assayed between PBS and 40 mg/kg group at 72 hours after the last injection. (C) The weights of heart, liver, spleen, lung, and kidney between PBS and 40 mg/kg group were monitored. (D) Major organs were harvested from PBS and 40 mg/kg groups for H&E staining and histological analysis. No abnormal histological conditions (inflammation, necrosis, or structure changes) between groups were observed. Data were presented as mean ± SEM. ^*^*p* < 0.05, ns, not significant.

**
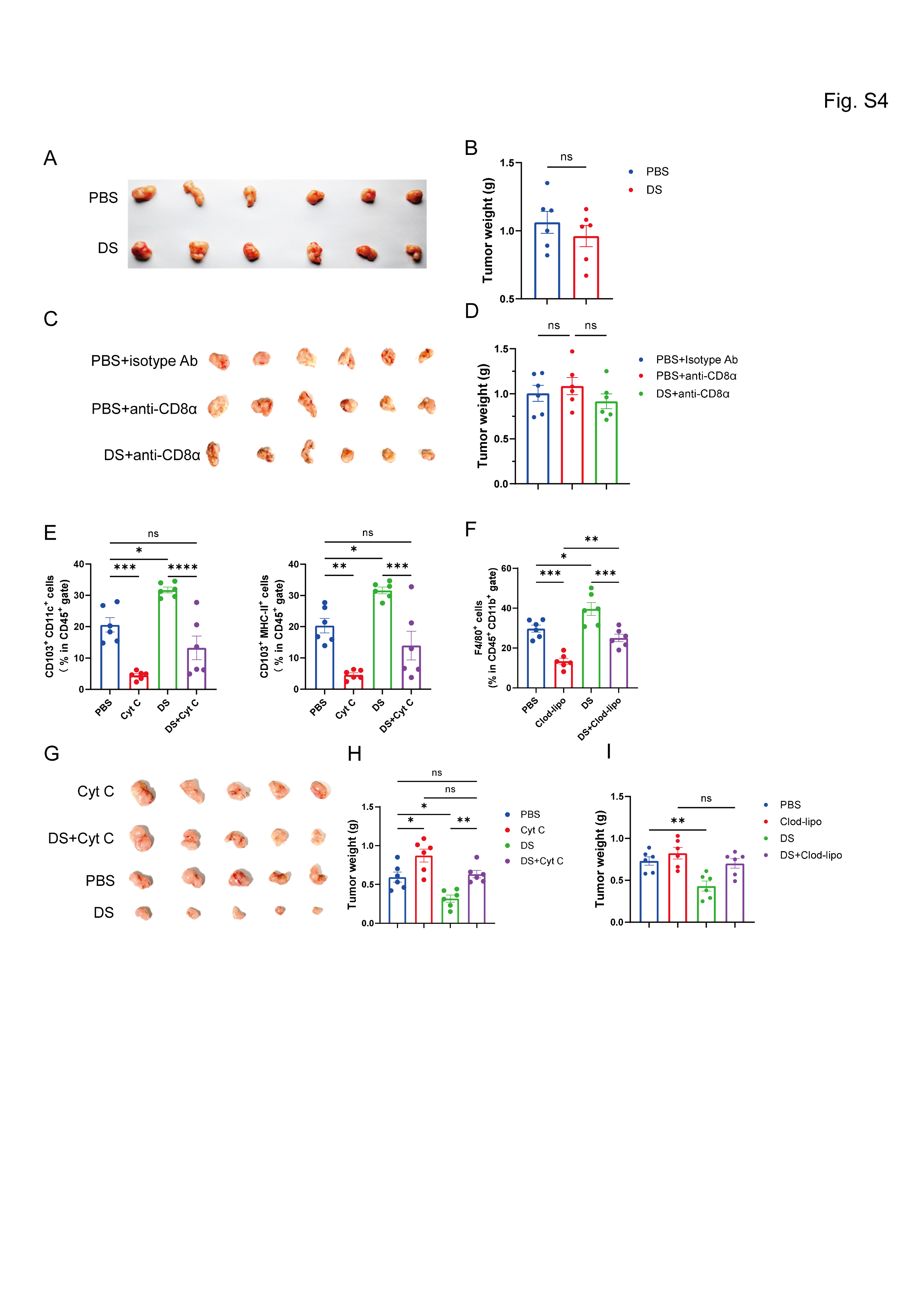
Fig S4****.** **DS inhibits tumor growth and stimulates antitumor immunity.** (A, B) Balb/c-null mice with subcutaneous MC38 tumor (n=6) were *i.p.* injected with PBS or DS (20 mg/kg). The tumors were observed ex vivo from mice (A). The tumor weight was measured at day 14 (B). (C, D) C57BL/6 mice bearing MC38 tumor (n=6) were treated with PBS, anti-CD8α neutralizing antibody or isotype antibody (100 μg per mouse, *i.v.*), and/or DS (20 mg/kg, *i.p.*). The tumors were ex vivo observed from mice (C), and the tumor weight was measured at day 14 (D). (E) C57BL/6 mice bearing MC38 tumor (n=6) were treated with PBS, Cyt c that deplete DCs, and/or DS (20 mg/kg, *i.v.*). The proportion of CD103^+^ CD11C^+^ cells and CD103^+^ MHC-II^+^ cells in TME from each group were measured by flow cytometry analysis. (F) C57BL/6 mice bearing MC38 tumor (n=6) were treated with PBS, clod-lipo that deplete macrophages, and/or DS (20 mg/kg, *i.p.*). The proportion of F4/80^+^ cells in CD45^+^ CD11b^+^ gate in TME from each group were measured by flow cytometry analysis. (G, H) C57BL/6 mice bearing MC38 tumor (n=6) were treated with PBS, Cyt c that deplete DCs, and/or DS (20 mg/kg, *i.v.*). The tumors were ex vivo observed from mice (G), and the tumor weight was measured at day 20 (H). (I) C57BL/6 mice bearing MC38 tumor (n=6) were treated with PBS, clod-lipo that deplete macrophages, and/or DS (20 mg/kg, *i.p.*). The tumor weight was measured at day 20. Statistical significance was analyzed by unpaired Student’s *t*-test (B), or one‐way ANOVA test (D-F, H, and I). ^*^*p* < 0.05, ^**^*p* < 0.01, ^***^*p* < 0.001, ns, not significant.

**
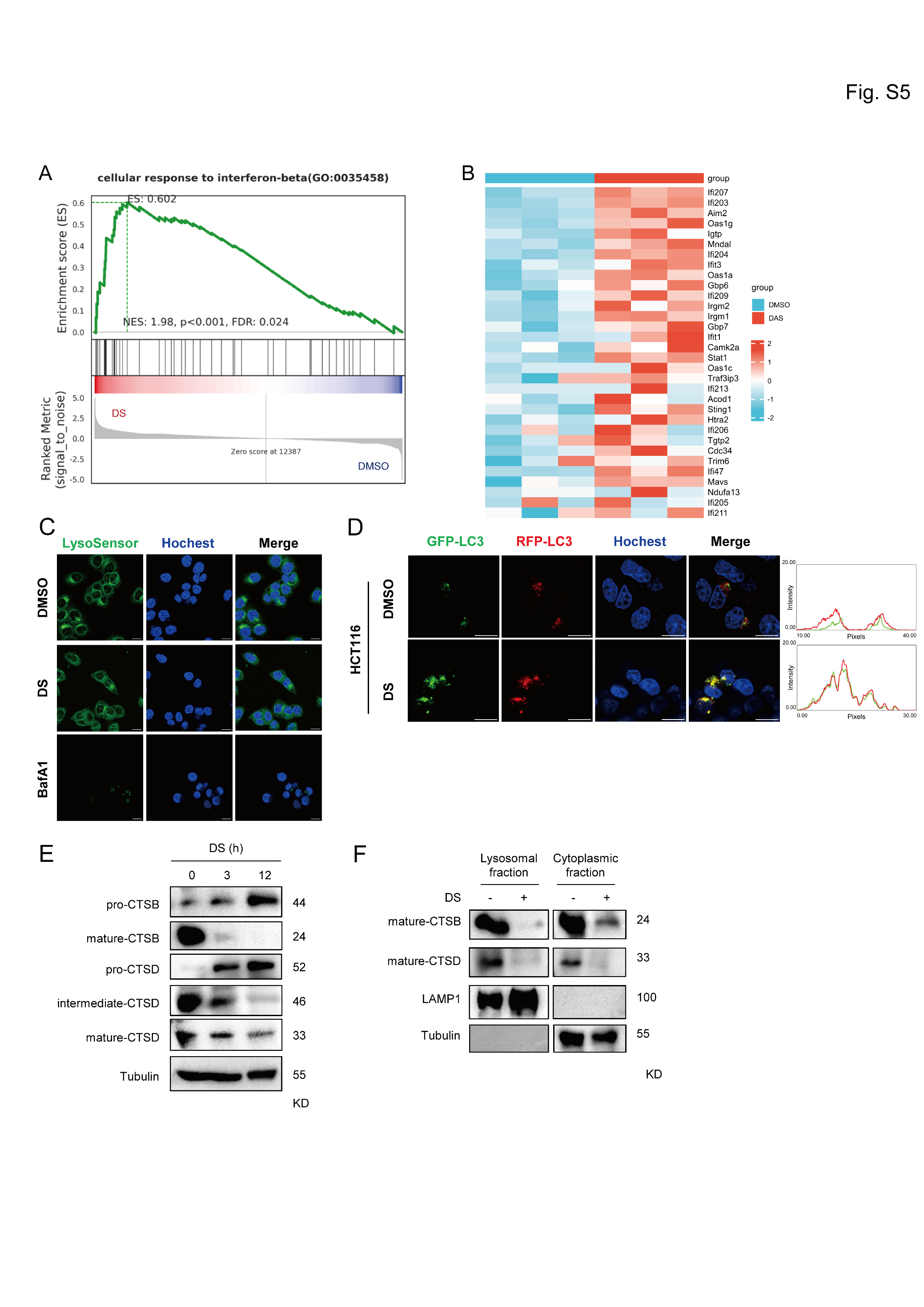
Fig S5. DS-mediated lysosomal inhibition induces IFN-I activation.** (A) According to RNA-seq data, GSEA analysis enriched DS into GO cellular response to interferon-beta. NES, normalized enrichment score; NOM, nominal; FDR, false discovery rate. (B) Genes of response to interferon-beta (GO: 0035458) differentially expressed in MC38 cells treated with DMSO or DS (20 μM) analyzed by RNA-seq and shown in the heatmap. (C) Immunofluorescence assay analysis of the LysoSensor Green staining in HCT116 cells treated with PBS or DS (20 μM) for 6 hours. The nuclei were stained with Hoechst 33342. 200 nM BafA1-treated cells were used as positive controls. Scale bars, 10 μm. (D) HCT116 cells were transfected with RFP-GFP-LC3 plasmid, followed by PBS or DS (20 μM) treatment for 12 hours. Representative fluorescent images are visualized with confocal microscopy (scale bars, 10 μm). The intensity profiles of GFLC3 and RFP-LC3 are shown in the right panel. (E) MC38 cells were treated with 20 μM DS for 3 or 12 hours, the precursor and the mature form of CTSB and CTSD were determined by immunoblotting. (F) Immunoblotting detecting the cytosolic and lysosomal distribution of mature CTSB and CTSD levels in MC38 cells treated with 20 μM DS for 12 hours. Lysosomal-associated membrane protein 1 (LAMP1) was as a lysosomal marker, Tubulin served as the cytosolic marker.

**
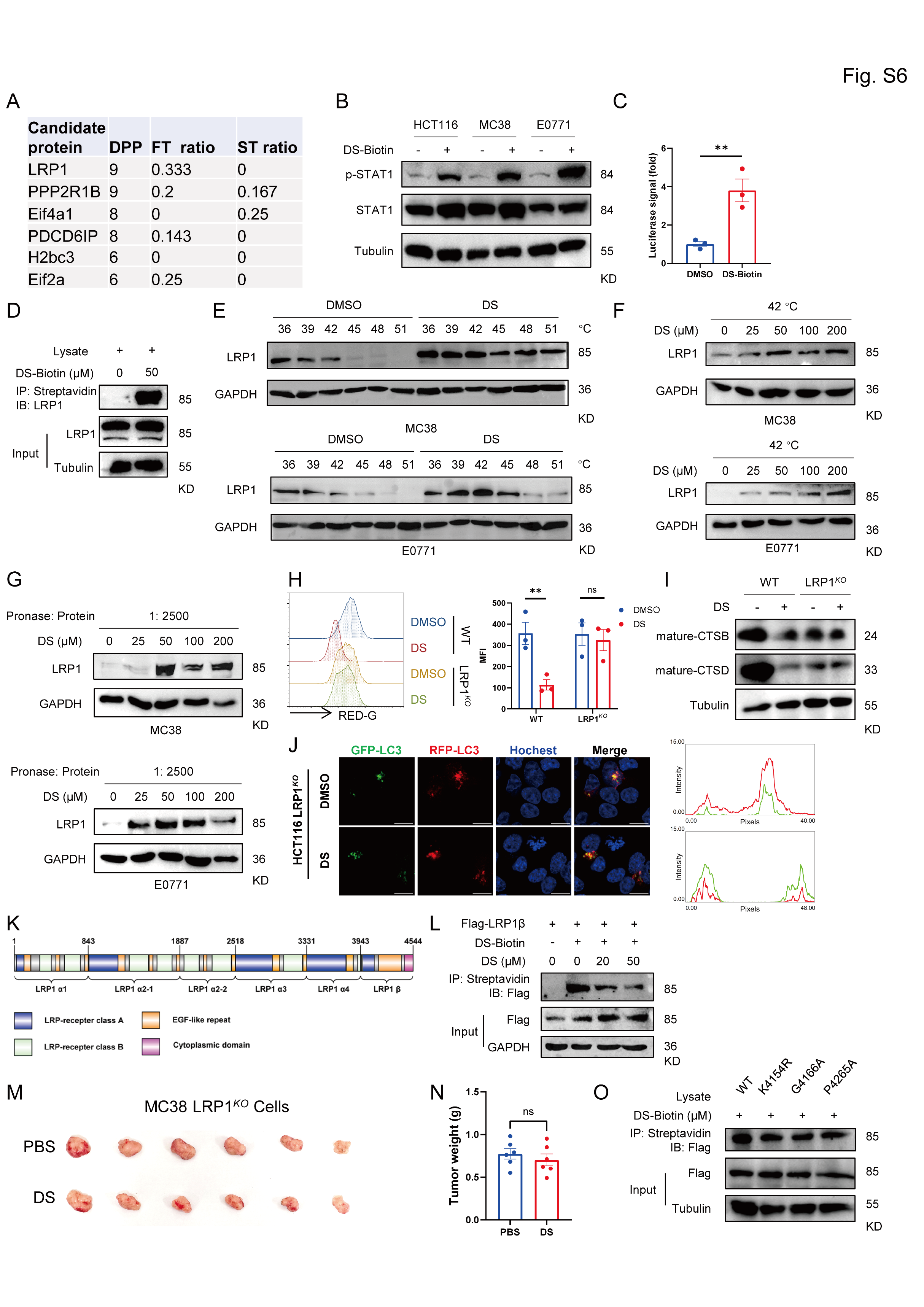
Fig S6****. LRP1 is the direct target protein of DS.** (A) LiP-SMap predicted top candidate target proteins of DS. Delta-Preference peptide (DPP) = Up_Full+Down_semi-Up_semi-Down_full > 5, preference full-trypsin peptide ratio (FT ratio) = Down_full/Up_full < 0.5, preference semi-trypsin peptide ratio (ST ratio) = Up_semi/Down_semi < 0.5. (B) Immunoblotting analysis of the phosphorylation level of STAT1 in HCT116 cells, MC38 cells and E0771 cells after treatment with DMSO or DS-Biotin (20 µM) for 24 hours. (C) HCT116 cells were transfected with IFN-β-Luc reporter plasmid and treated with DS-biotin (20 μM) for 24 hours, The activity of IFN-β was measured by dual luciferase reporter gene assay. (D) Immunoblotting analyzed the streptavidin-agarose precipitated LRP1 from HCT116 cells incubated with DS-Biotin at 4°C overnight. (E) CETSA analysis of the binding of DS to LRP1 in MC38 or E0771 cells. (F) The stability of LRP1 engagement by indicated concentrations of DS at 42°C was determined by CETSA. (G) DARTS assay determined the stability of LRP1 engagement by indicated concentrations of DS when the ratio of Pronase to protein was 1:2500 in MC38 and E0771 cells. (H) HCT116 WT or LRP1*^KO^* cells were treated with DS (20 μM) for 6 hours before being incubated with DQ-Red BSA (10 μg/ml) for 2 hours. Red fluorescence stimulated by green laser (RED-G) was detected by flow cytometry analysis. Quantification of mean fluorescence intensity (MFI) of was shown. (I) HCT116 WT cells and HCT116 LRP1*^KO^* cells were treated with 20 μM DS for 12 hours, the mature form of CTSB and CTSD were determined by immunoblotting. (J) HCT116 LRP1*^KO^* cells were treated with PBS or DS (20 μM) for 12 hours after transfected with RFP-GFP-LC3B reporter for 48 hours. Fluorescence images of the cells and analysis of the number of autophagosomes and autolysosomes (scale bars, 10 μm). (K) Schematic representation of LRP1 truncations. (L) 293T cells were transfected with Flag-LRP1β for 48 hours, then the lysates were incubated with indicated concentrations of DS-biotin in the absence or presence of a two-fold excess of unlabeled DS at 4°C overnight, the mixtures were precipitated by streptavidin-agarose and immunoblotted with Flag antibody. (M, N) C57BL/6 mice with subcutaneous LRP1*^KO^* MC38 tumor (n=6) were *i.p.* injected with PBS or DS (20 mg/kg), the tumors were *ex vivo* observed from mice (M). The tumor weight was measured at day 24 (N). (O) 293T cells were transfected with LRP1-WT and LRP1-mutant plasmids for 48 h, cell lysates were incubated with RA-biotin at 25°C for 2 hours, followed by pull-down with streptavidin-agarose; the precipitates were then immunoblotted by Flag antibody. Data were presented as mean ± SEM. Statistical significance was analyzed by unpaired Student’s *t*-test (C, H, and N). ^*^*p* < 0.05, ^**^*p* < 0.01, ^***^*p* < 0.001, ns, not significant.

**
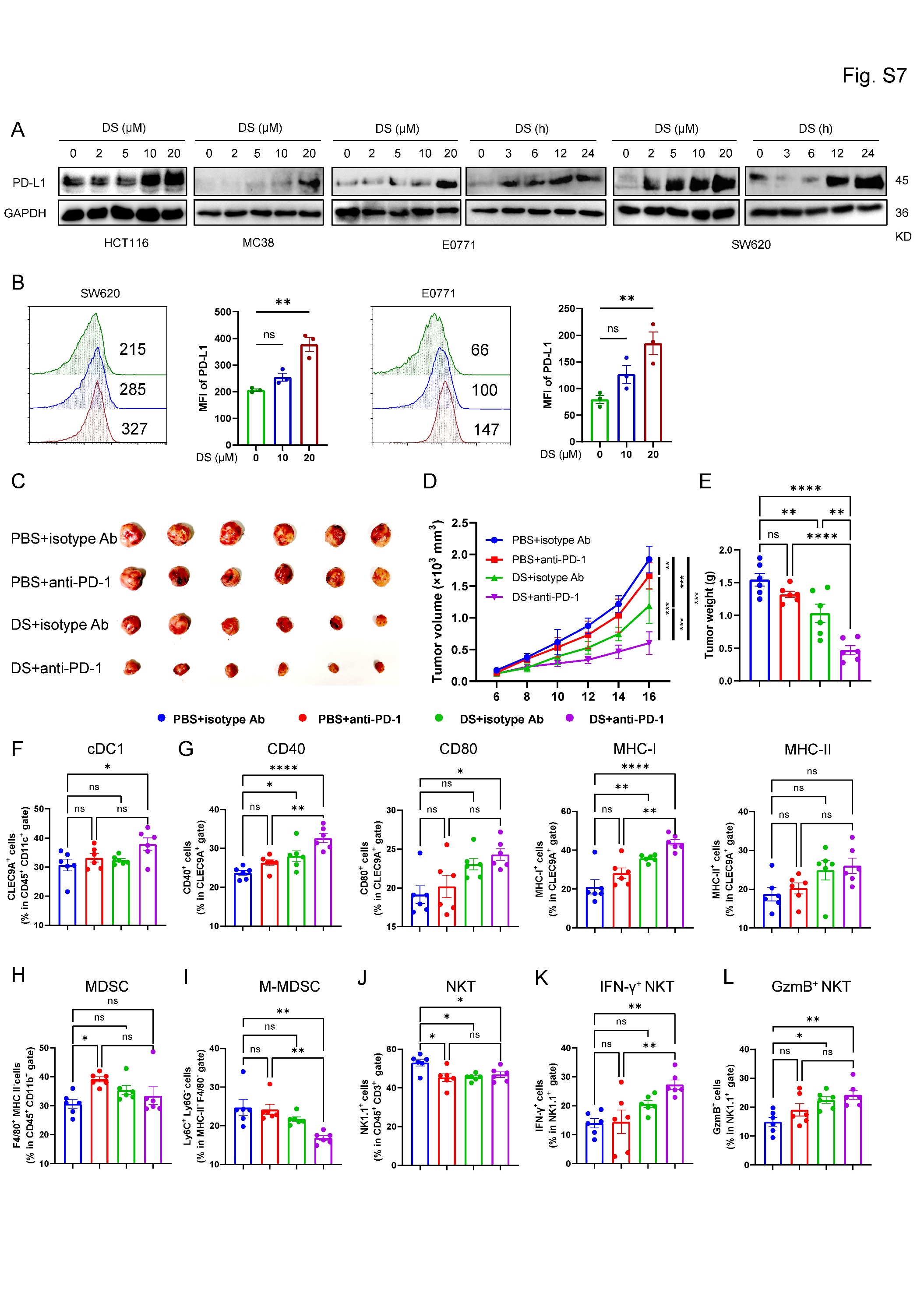
**

**Fig S7****. DS induces immune cell infiltration and potentiates efficacy of anti-PD-1 therapy.** (A) HCT116, MC38, SW620 and E0771 cells were treated with DS at the indicated concentrations for 24 hours, or treated with DS (20 μM) for indicated time points, the expression levels of PD-L1 were detected by immunoblotting. (B) SW620 and E0771 cells were treated with DS at the indicated concentrations for 24 hours, or treated with DS (20 μM) for indicated time points, and the plasma membrane levels of PD-L1 were detected by flow cytometry analysis. Quantification of mean fluorescence intensity (MFI) of was shown. (C-E) C57BL/6 mice bearing E0771 tumor (n=6) were treated with PBS, anti-PD-1 antibody (100 μg per mouse), DS (20 mg/kg), or the combination. the tumors were observed *ex vivo* from mice (C), and the tumor volume (D) and tumor weight (E) were monitored. (F-L) C57BL/6 mice bearing MC38 tumor (n=6) were treated with PBS, anti-PD-1 antibody (100 μg per mouse), DS (20 mg/kg), or the combination. The proportion of cDC1s (F), surface expression levels of CD40, CD80, MHC-I and MHC-II (G) on CLEC9A^+^ cells in TME from each group were determined by flow cytometry analysis. The proportion of MDSCs (H) and M-MDSCs (I), NKT cells (J), and the effector molecules IFN-γ^+^ cells (K) and GzmB^+^ cells (L) in NKT cells gate in TME from each group were determined by flow cytometry analysis. The experiments (A and B) were repeated three times independently with similar results. Data were presented as mean ± SEM. Statistical significance was analyzed by one‐way ANOVA test (B, E-L), or two‐way ANOVA test (D). ^*^*p* < 0.05, ^**^*p* < 0.01, ^***^*p* < 0.001, ns, not significant.


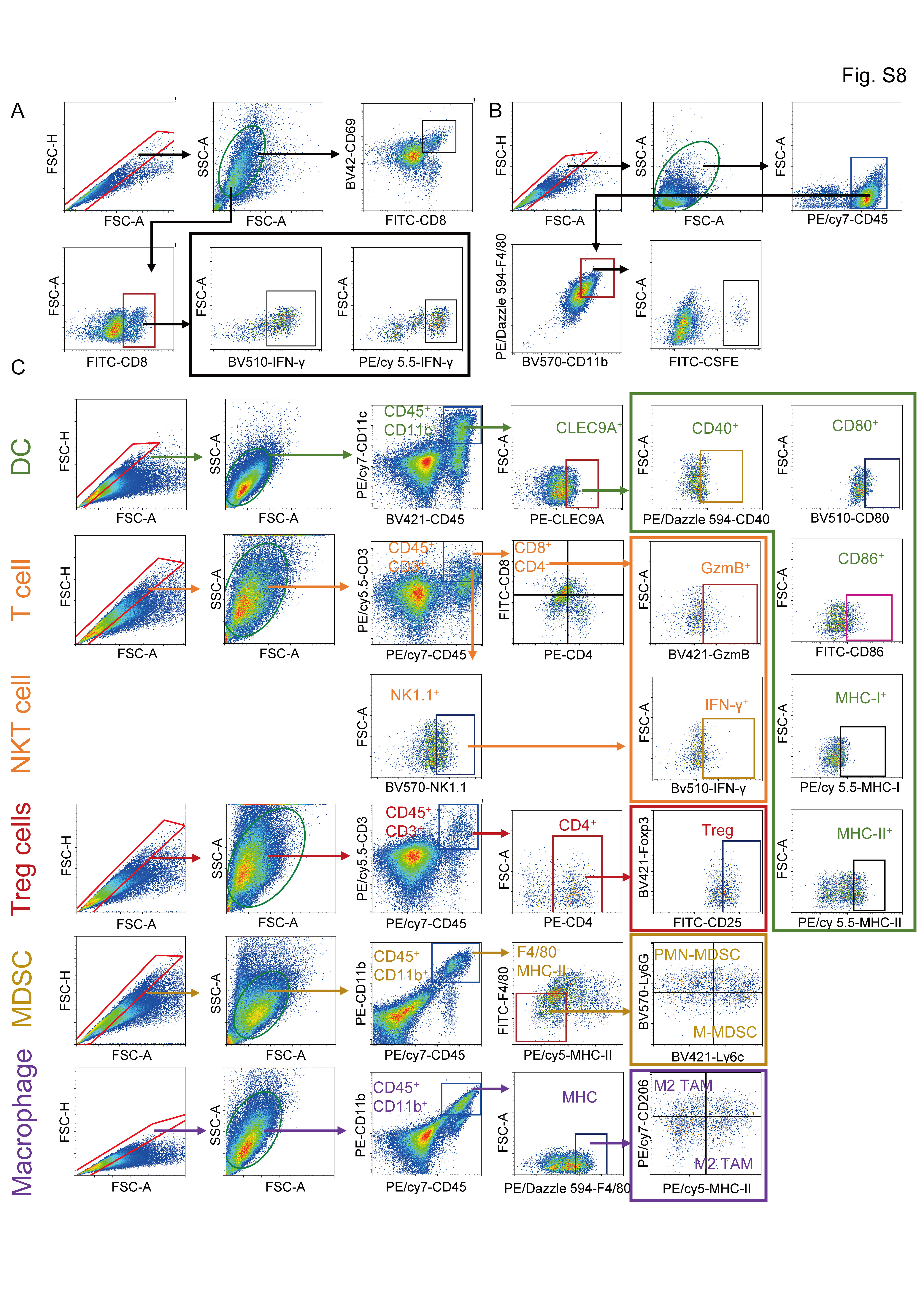


**Fig S8. Gating strategies for flow cytometry analysis.** (A) Gating strategy for CD8^+^ T cell, CD69, IFN-γ and GzmB in tumor cells-BMDCs-T cells co-culture assay. (B) Gating strategy for CD45^+^ CD11b^+^ F4/80^+^ cells, CFSE in tumor cells-BMDM co-culture assay. (C) Flow cytometry gating strategies for analysis of tumor-infiltrating cDC1s, CD8^+^ T cells, NKT cells, Tregs, MDSCs, and macrophages were shown.

**Table S1** Reagents and commercial assay kits used in this study

| COMMERCIAL KITS | SOURCE | IDENTIFIER |
| --- | --- | --- |
| Natural Product Library | Selleck | Cat# L1400 |
| Dual-Luciferase Reporter Assay System | Promega | Cat# E1960 |
| TRNzol Universal Reagent | TIANGEN | Cat# DP424 |
| Fast Site-Directed Mutagenesis Kit | TIANGEN | Cat# KM101 |
| UniPeak U+ One Step RT-qPCR SYBR Green Kit | Vazyme | Cat# Q226-01 |
| Mouse IFN-beta Quantikine ELISA Kit | R&D Systems | Cat# MIFNB0 |
| Mouse CXCL10/IP-10/CRG-2 ELISA Kit | R&D Systems | Cat# DY466-05 |
| Human IFN-beta Quantikine ELISA Kit | R&D Systems | Cat# DIFNB0 |
| Human CXCL10/IP-10 Quantikine ELISA Kit | R&D Systems | Cat# DIP100 |
| Lysosome Extraction Kit | Sigma-Aldrich | Cat# LYSISO1 |
| CHEMICALS AND RECOMBINANT PROTEINS |  |  |
| DMEM | Gibco | Cat# C11995500BT |
| RPMI1640 | Gibco | Cat# C118875500BT |
| Fetal Bovine Serum | Gibco | Cat# 25200-056 |
| Daurisoline | MedChemExpress | Cat# HY-N0221 |
| Bafilomycins | MedChemExpres | Cat# HY-100558 |
| Chloroquine | MedChemExpress | Cat# HY-17589A |
| Recombinant Mouse GM-CSF | Peprotech | #315-03 |
| Recombinant Mouse IL-4 | Peprotech | #214-14 |

**Table S2** Antibodies used for immunoblotting.

| ANTIBODY | SOURCE | IDENTIFIER |
| --- | --- | --- |
| GAPDH Rabbit mAb | Cell Signaling Technology | Cat# 5174 |
| β-Tubulin Antibody | Cell Signaling Technology | Cat# 2146 |
| TBK1/NAK Rabbit mAb | Cell Signaling Technology | Cat# 38066 |
| Phospho-TBK1/NAK (Ser172) Rabbit mAb | Cell Signaling Technology | Cat# 5483 |
| Stat1 Antibody | Cell Signaling Technology | Cat# 9172 |
| Phospho-Stat1 (Ser727) Antibody | Cell Signaling Technology | Cat# 9177 |
| LRP1 Rabbit pAb | Proteintech | Cat# 26106-1-AP |
| DYKDDDDK Tag Mouse mAb | Cell Signaling Technology | Cat# 8146 |
| Cathepsin B Rabbit mAb | Cell Signaling Technology | Cat# 31718 |
| Cathepsin D Antibody | Santa Cruz Biotechology | Cat# sc-6486 |
| LC3 Rabbit pAb | MBL | Cat# PM036 |
| SQSTM1/p62 Antibody | Cell Signaling Technology | Cat# 5114 |
| LAMP1 Rabbit mAb | Cell Signaling Technology | Cat# 9091 |
| STING Rabbit mAb | Cell Signaling Technology | Cat# 13647 |
| Phospho-STING (Ser365) Rabbit mAb | Cell Signaling Technology | Cat# 72971 |
| IRF-3 Rabbit mAb | Cell Signaling Technology | Cat# 4302 |
| Phospho-IRF-3 (Ser396) Rabbit mAb | Cell Signaling Technology | Cat# 4947 |
| PD-L1 (E1L3N^®^) XP^®^ Rabbit mAb | Cell Signaling Technology | Cat# 13684 |
| PD-L1 (D4H1Z) Rabbit mAb | Cell Signaling Technology | Cat# 60475 |

**Table S3** Antibodies used for flow cytometry, IF and IHC analysis

| ANTIBODY | SOURCE | IDENTIFIER |
| --- | --- | --- |
| PE/Cyanine7 anti-mouse CD11c antibody | Biolegend | Cat# 117317 |
| FITC anti-mouse CD103 antibody | Biolegend | Cat# 121419 |
| PE/Cyanine5 anti-mouse CD40 antibody | Biolegend | Cat# 124617 |
| PE/Dazzle™ 594 anti-mouse CD40 antibody | Biolegend | Cat# 124630 |
| FITC anti-mouse CD86 antibody | Biolegend | Cat# 159220 |
| PE anti-mouse CD370 (CLEC9A, DNGR1) antibody | Biolegend | Cat# 143504 |
| PE anti-mouse CD80 antibody | Biolegend | Cat# 104708 |
| Brilliant Violet 510™ anti-mouse CD80 antibody | Biolegend | Cat# 104741 |
| PerCP/Cyanine5.5 anti-mouse H-2Kb antibody | Biolegend | Cat# 116516 |
| Brilliant Violet 605™ anti-mouse I-A/I-E antibody | Biolegend | Cat# 107639 |
| PE/Cyanine5 anti-mouse I-A/I-E antibody | Biolegend | Cat# 107612 |
| PE anti-mouse H-2Kb SIINFEKL antibody | Biolegend | Cat# 141603 |
| PE/Cyanine7 anti-mouse CD45 antibody | Biolegend | Cat# 157205 |
| Brilliant Violet 421™ anti-mouse CD45 antibody | Biolegend | Cat# 103133 |
| PerCP/Cyanine5.5 anti-mouse CD3 antibody | Biolegend | Cat# 100217 |
| FITC anti-mouse CD8a antibody | Biolegend | Cat# 100705 |
| PE anti-mouse CD4 antibody | Biolegend | Cat# 100407 |
| Brilliant Violet 421™ anti-human/mouse Granzyme B Recombinant antibody | Biolegend | Cat# 396414 |
| PerCP/Cyanine5.5 anti-human/mouse Granzyme B Recombinant antibody | Biolegend | Cat# 372212 |
| Brilliant Violet 510™ anti-mouse IFN-γ antibody | Biolegend | Cat# 505841 |
| PE/Dazzle™ 594 anti-mouse Ki-67 antibody | Biolegend | Cat# 652428 |
| Brilliant Violet 570™ anti-mouse NK-1.1 antibody | Biolegend | Cat# 108733 |
| PE anti-mouse/human CD11b antibody | Biolegend | Cat# 101207 |
| FITC anti-mouse F4/80 antibody | Biolegend | Cat# 123107 |
| PE/Cyanine7 anti-mouse CD206 (MMR) antibody | Biolegend | Cat# 141720 |
| FITC anti-mouse CD25 antibody | Biolegend | Cat# 101907 |
| Brilliant Violet 421™ anti-mouse FOXP3 antibody | Biolegend | Cat# 126419 |
| Brilliant Violet 421™ anti-mouse Ly-6C antibody | Biolegend | Cat# 128031 |
| Brilliant Violet 570™ anti-mouse Ly-6G antibody | Biolegend | Cat# 127629 |
| Brilliant Violet 421™ anti-mouse CD69 antibody | Biolegend | Cat# 104527 |
| PE anti-mouse CD274 (B7-H1, PD-L1) antibody | Biolegend | Cat# 124308 |
| PE anti-human CD274 (B7-H1, PD-L1) antibody | Biolegend | Cat# 329706 |

**Table S4** Primers for RT-qPCR

| Mouse IFNB1 forward | ATGGAGATGACGGAGAAGATGC |
| --- | --- |
| Mouse IFNB1 reverse | TTCAGAAACACTGTCTGCTGGT |
| Mouse CXCL10 forward | TCATTTTCTGCCTCATCCTGCT |
| Mouse CXCL10 reverse | TCTGCAAGCTGAAGGGATTTCT |
| Mouse GAPDH forward | TGGCCTCCAAGGAGTAAGAAAC |
| Mouse GAPDH reverse | ATTCAAGAGAGTAGGGAGGGCT |
| Human CXCL10 forward | CCACGTGTTGAGATCATTGCT |
| Human CXCL10 reverse | TGCATCGATTTTGCTCCCCT |
| Human IFNβ forward | AGTAGGCGACACTGTTCGTG |
| Human IFNβ reverse | GCCTCCCATTCAATTGCCAC |
| Human GAPDH forward | TGCACCACCAACTGCTTAGC |
| Human GAPDH reverse | GGCATGGACTGTGGTCATGAG |
| Mouse Tap1 forward | GGACTTGCCTTGTTCCGAGAG |
| Mouse Tap1 reverse | GCTGCCACATAACTGATAGCGA |
| Mouse Tap2 forward | CTGGCGGACATGGCTTTACTT |
| Mouse Tap2 reverse | CTCCCACTTTTAGCAGTCCCC |
| Mouse Earp1 forward | TAATGGAGACTCATTCCCTTGGA |
| Mouse Earp1 reverse | AAAGTCAGAGTGCTGAGGTTTG |
| Mouse B2m forward | TTCTGGTGCTTGTCTCACTGA |
| Mouse B2m reverse | CAGTATGTTCGGCTTCCCATTC |

**Table S5** sgRNA sequence for knocking out the indicated proteins.

| sgRNA: Anxa5 F | CACCGATCAATTTCAGGTACCGATG |
| --- | --- |
| sgRNA: Anxa5 R | AAACCATCGGTACCTGAAATTGATC |
| sgRNA: Eif2a F | CACCGGTTAAGATATGCTCACCATC |
| sgRNA: Eif2a R | AAACGATGGTGAGCATATCTTAACC |
| sgRNA: Eif4a1 F | CACCGCCACCTCGACCGATTCTGAG |
| sgRNA: Eif4a1 R | AAACCTCAGAATCGGTCGAGGTGGC |
| sgRNA: H2bc3 F | CACCGCCAGCTCCAAGTGAGCTCGT |
| sgRNA: H2bc3 R | AAACACGAGCTCACTTGGAGCTGGC |
| sgRNA: Hnrnpk F | CACCGCTGTTGGGACATACCGCTCG |
| sgRNA: Hnrnpk R | AAACCGAGCGGTATGTCCCAACAGC |
| sgRNA: Lgals1 F | CACCGGTCTCACCTCTTGGCGTCCG |
| sgRNA: Lgals1 R | AAACCGGACGCCAAGAGGTGAGACC |
| sgRNA: Lrp1 F | CACCGCGTGGACCAGACTCGCCCAG |
| sgRNA: Lrp1 R | AAACCTGGGCGAGTCTGGTCCACGC |
| sgRNA: Myl6 F | CACCGGACCCTTACCCAGTGTGACT |
| sgRNA: Myl6 R | AAACAGTCACACTGGGTAAGGGTCC |
| sgRNA: Naa20 F | CACCGGCGGCGCAATGACCACGCTC |
| sgRNA: Naa20 R | AAACGAGCGTGGTCATTGCGCCGCC |
| sgRNA: Pcbp2 F | CACCGCAACACCGGATTCAGTGGTA |
| sgRNA: Pcbp2 R | AAACTACCACTGAATCCGGTGTTGC |
| sgRNA: Pdcd6ip F | CACCGTTACCCGGGATATCCTGGAT |
| sgRNA: Pdcd6ip R | AAACATCCAGGATATCCCGGGTAAC |
| sgRNA: Ppp2cb F | CACCGACATGAGGCTCTCCACGACG |
| sgRNA: Ppp2cb R | AAACCGTCGTGGAGAGCCTCATGTC |
| sgRNA: Ppp2r1b F | CACCGCTGGCGGGTTCGATATATGG |
| sgRNA: Ppp2r1b R | AAACCCATATATCGAACCCGCCAGC |
| sgRNA: Rhoa F | CACCGTCGTGTGCTCGTCATTCCGA |
| sgRNA: Rhoa R | AAACTCGGAATGACGAGCACACGAC |
| sgRNA: Rplp2 F | CACCGGACGTAGCGCATGCTAAATG |
| sgRNA: Rplp2 R | AAACCATTTAGCATGCGCTACGTCC |
| sgRNA: Sin3a F | CACCGCGTATCCGGAAGTGTCAACG |
| sgRNA: Sin3a R | AAACCGTTGACACTTCCGGATACGC |
| sgRNA: Timm8a1 F | CACCGCCATCCCGGCACGACCACGC |
| sgRNA: Timm8a1 R | AAACGCGTGGTCGTGCCGGGATGGC |
| sgRNA: Tomm22 F | CACCGACGACGACGACGAGGTGCTA |
| sgRNA: Tomm22 R | AAACTAGCACCTCGTCGTCGTCGTC |
| sgRNA: SQSTM1 F | CACCGCGTGAACGACGCCATAACCG |
| sgRNA: SQSTM1 R | AAACCGGTTATGGCGTCGTTCACGC |
| sgRNA: TAX1BP1 F | CACCGCCGCCGGACTACGACCAGCA |
| sgRNA: TAX1BP1 R | AAACTGCTGGTCGTAGTCCGGCGGC |
| sgRNA: NBR1 F | CACCGGGTAAGCAGTCCGCGCTCGG |
| sgRNA: NBR1 R | AAACCCGAGCGCGGACTGCTTACCC |
| sgRNA: OPTN F | CACCGGAGTGAATCGGAATACTGCG |
| sgRNA: OPTN R | AAACCGCAGTATTCCGATTCACTCC |
| sgRNA: AZI2 F | CACCGTGCTCTTCTCTCGACAAGCT |
| sgRNA: AZI2 R | AAACAGCTTGTCGAGAGAAGAGCAC |

**Table S6** Plasmids used in this study

| Plasmid | SOURCE | IDENTIFIER |
| --- | --- | --- |
| LentiCrisprV2 | Addgene | #98290 |
| psPAX2 | Addgene | #12260 |
| VSVG | Addgene | #1733 |
| FN-β_Pgl3 | Addgene | #102597 |
| pRL-TK | Beyotime | #D2760 |
| pCI-neo-mOVA | Addgene | #25099 |
| mCherry-EGFP-LC3 | Preserved in our laboratory | - |
| EGFP-LC3 | Preserved in our laboratory | - |
